# Chemical regulators of epithelial plasticity reveal a nuclear receptor pathway controlling myofibroblast differentiation

**DOI:** 10.1038/srep29868

**Published:** 2016-07-19

**Authors:** Jon M. Carthy, Martin Stöter, Claudia Bellomo, Michael Vanlandewijck, Angelos Heldin, Anita Morén, Dimitris Kardassis, Timothy C. Gahman, Andrew K. Shiau, Marc Bickle, Marino Zerial, Carl-Henrik Heldin, Aristidis Moustakas

**Affiliations:** 1 Ludwig Institute for Cancer Research, Science for Life Laboratory, Uppsala University, Box 595, Biomedical Center, SE-751 24 Uppsala, Sweden; 2 Max Planck Institute of Molecular Cell Biology and Genetics, Dresden, Germany; 3 Department of Medical Biochemistry and Microbiology, Science for Life Laboratory, Uppsala University, Box 582, Biomedical Center, SE-751 23 Uppsala, Sweden; 4 Department of Biochemistry, University of Crete Medical School, 71003 Heraklion, Crete, Greece; 5 Small Molecule Discovery Program, Ludwig Institute for Cancer Research, La Jolla, CA 92093, USA

## Abstract

Plasticity in epithelial tissues relates to processes of embryonic development, tissue fibrosis and cancer progression. Pharmacological modulation of epithelial transitions during disease progression may thus be clinically useful. Using human keratinocytes and a robotic high-content imaging platform, we screened for chemical compounds that reverse transforming growth factor β (TGF-β)-induced epithelial-mesenchymal transition. In addition to TGF-β receptor kinase inhibitors, we identified small molecule epithelial plasticity modulators including a naturally occurring hydroxysterol agonist of the liver X receptors (LXRs), members of the nuclear receptor transcription factor family. Endogenous and synthetic LXR agonists tested in diverse cell models blocked α-smooth muscle actin expression, myofibroblast differentiation and function. Agonist-dependent LXR activity or LXR overexpression in the absence of ligand counteracted TGF-β-mediated myofibroblast terminal differentiation and collagen contraction. The protective effect of LXR agonists against TGF-β-induced pro-fibrotic activity raises the possibility that anti-lipidogenic therapy may be relevant in fibrotic disorders and advanced cancer.

Epithelia compose a large part of human organs including the starting embryonic cell type. During embryogenesis, tissue homeostasis and disease pathogenesis, epithelia are remodelled locally by generating mesenchymal derivatives that migrate and establish new tissues in the embryonic cavities or assist in tissue wound healing after birth^1^. Prolonged tissue wounding with chronic inflammation causes mesenchymal constituents to contribute to tissue fibrosis and cancer progression instead of permitting physiological healing^1^. Under such developmental and pathological circumstances the process of epithelial-mesenchymal transition (EMT), a transient and reversible change in epithelial differentiation that generates transitory mesenchymal cell types, becomes important^1^. EMT is induced by developmental growth factor pathways, among which transforming growth factor β (TGF-β) has a prominent role^2^. EMT generates a spectrum of transitory cell phenotypes defined based on molecular markers that include transcription factors, cell-cell junctional proteins, cytoskeletal and extracellular matrix proteins and secreted cytokines^3^,^4^.

TGF-β not only induces EMT but also negatively regulates epithelial proliferation, induces epithelial cell death, and regulates many non-epithelial cell types in embryos and in adult tissues^5^. The signalling pathway of TGF-β is frequently misregulated in human diseases, including cancer and tissue fibrosis, a hallmark manifestation of TGF-β hyperactivity^6^. By binding to its type II and type I serine/threonine kinase receptors, TGF-β activates a signalling cascade that involves Smad proteins and various branches of protein kinases, including mitogen activated protein kinases (MAPKs) and small GTPases, which coordinately affect gene expression to manifest the biological effects of this growth factor^5^. To catalyse EMT, TGF-β causes disassembly of cell-cell junctional complexes, remodels microfilaments and intermediate filaments, induces large amounts of extracellular matrix biomolecules, including fibronectin, and causes secretion of other cytokines and chemokines^2^. Furthermore, prolonged TGF-β activity in a given epithelial tissue is usually associated with the accumulation of newly deposited matrix, terminal differentiation of myofibroblasts and recruitment of immune cells that contribute to the fibrotic phenotype^7^. Myofibroblasts, key cell types of the fibrotic microenvironment, can be derived from many sources including interstitial fibroblast progenitors, epithelial cells via EMT or endothelial cells via endothelial-mesenchymal transition^7^,^8^. Myofibroblasts generate tissue contractility which is catalysed by specialised α smooth muscle actin (αSMA) microfilaments and tight associations between the cytoskeleton, integrin family receptors and matrix proteins^8^. TGF-β activates transcriptional regulators, such as β-catenin and Smads, and MAPK signalling to control the activity of key transcription factors during myofibroblast differentiation, thus inducing the expression of *αSMA* and other fibrotic marker genes such as *collagens* and *fibronectin*^8^,^9^. Myofibroblast differentiation is also highly relevant to cancer; the tumour stroma contains cancer-associated fibroblasts (CAFs)^10^ that contribute to tumour cell invasiveness and immune surveillance suppression, mainly by secreting cytokines and chemokines, including TGF-β^11^.

Our aim was to identify small molecules and molecular pathways that control terminal EMT stages linked to myofibroblast differentiation by using prolonged exposure of epithelial cells to TGF-β, followed by treatment with compounds from a chemical library. The rationale being that such an approach might identify selective inhibitors of the pro-tumourigenic actions of TGF-β which, in contrast to known TGF-β receptor kinase inhibitors, would not inhibit the tumour suppressor pathways of TGF-β. Agonists of the nuclear receptors, liver X receptor (LXR) α and β, were among the epithelial plasticity modulators identified in our screen. Being major regulators of lipid metabolism, the LXRs have been targeted pharmacologically^12^,^13^. By studying further the crosstalk between TGF-β and LXR in the context of myofibroblast differentiation, we have uncovered a novel mechanism via which LXRα can counteract the pro-fibrotic action of TGF-β.

## Results

### Selection of biological parameters to analyse EMT modulators

**Figure 1:**
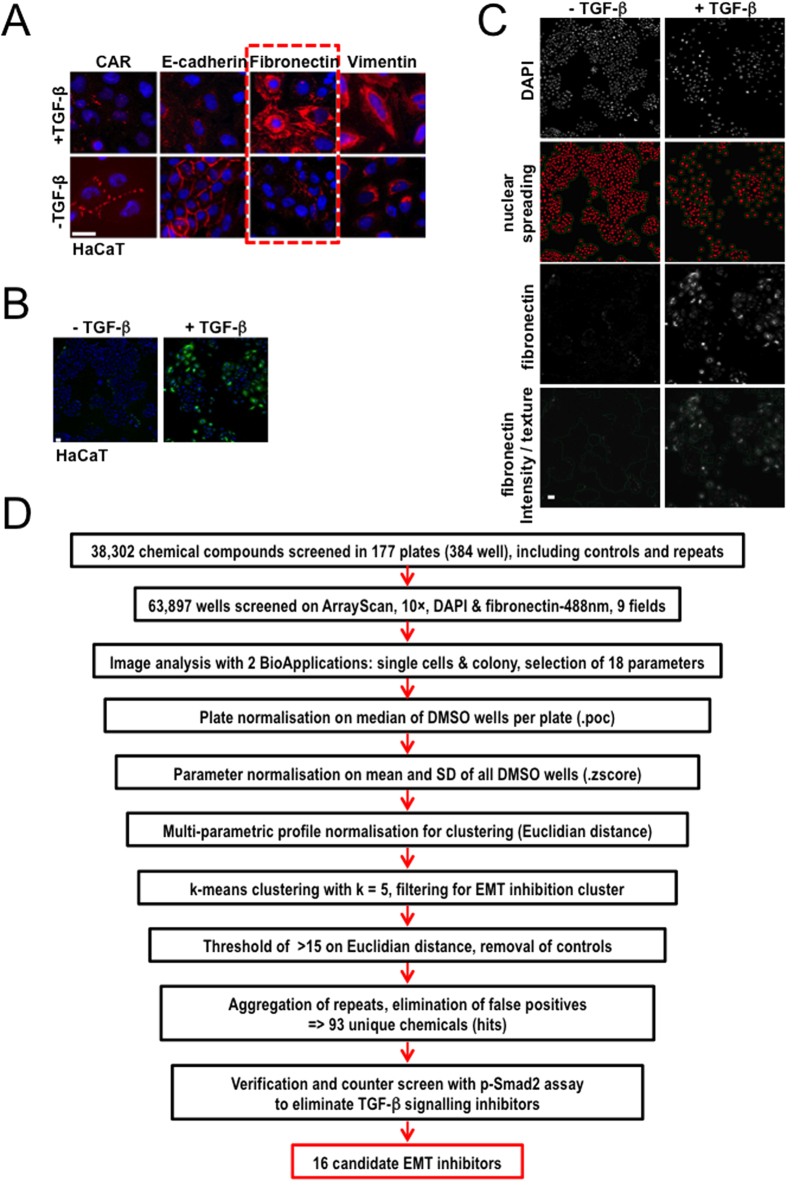
Overview of the high-content screen. (**A**) Immunofluorescence microscopy with the indicated antibodies after 72 h stimulation of HaCaT cells with 5 ng/ml TGF-β. A dotted frame marks fibronectin expression. (**B**) HaCaT cell images taken with ArrayScan. Nuclear distribution (blue) and fibronectin expression (green) changes upon TGF-β stimulation. (**C**) HaCaT cell image analysis using BioApplications. Displayed are changes in nuclear distribution (first row); nuclear spreading (second row) with two algorithms segmenting single nuclei (red spots) and groups of cells as colonies (green lines); fibronectin expression (third row) and fibronectin analysed as intensity and texture measurements within single cells and colonies (fourth row, green lines). White bars indicate 10 μm. (**D**) Schematic overview of data acquisition and analysis. 18 parameters selected (Fig. S3) from two individual image analysis algorithms (segmentation of nuclei as single cells and segmentation of grouped nuclei as colonies) were normalised plate-by-plate using the robust percentage-of-control (.poc) method and then normalised per parameter using the Z score (.zscore) method within the open-source software KNIME (HCS-Tools extensions). Clustering was performed with the k-means algorithm using k = 5 after normalisation of parameters using the Euclidian distance (phenotypic strength). The cluster containing EMT inhibitors was filtered for phenotypic strength greater than 15 and finally the hit list was condensed to 93 unique compounds that were hits in the primary screen. To remove compounds interfering with TGF-β signalling, a counter screen was performed using the p-Smad2 assay (see Fig. S4C,D) and 16 candidate EMT inhibitors were taken into further validation.

We screened several human cell models from diverse epithelial tissues (skin, lung, mammary) that exhibit TGF-β-mediated EMT in a time- and dose-dependent manner^14^. These included human immortalised keratinocytes HaCaT, human lung epithelial cells HPL1, human immortalised mammary epithelial cells HMLE, MCF-10A, a human mammary lumenal and a human mammary myoepithelial cell model. From these cell models, HaCaT keratinocytes, which were previously established to undergo EMT in response to TGF-β^15^,^16^, appeared most suitable for the high-throughput assay (Fig. 1A). Several EMT markers, including the tight junction protein coxsackie and adenovirus receptor (CAR), the adherens junction protein E-cadherin, the extracellular matrix protein fibronectin, the intermediate filament protein vimentin (Fig. 1A) and a few more (adaptor protein ZO-1, tight junction membrane proteins occludin and claudins, actin microfilaments), were analysed in these cells before and after stimulation by TGF-β. For several makers signal-to-noise ratios and Z′-factors^17^ were calculated. For the image analysis software the junctional protein markers, such as E-cadherin or ZO-1, were in general more difficult to quantify. Vimentin and actin staining revealed low signal-to-noise ratios. Taken together, we concluded that fibronectin (Fig. 1A, dashed box, Figs S1A and S2A) was the most reliable protein marker for a robotic microscope-based screen. Quantitative Z′-factor data convincingly showed that, for example, E-cadherin could not serve the same purpose (Fig. S2B). The choice of fibronectin is in agreement with a recently reported high-content screen preformed in a mammary epithelial cell model, which identified a set of kinase inhibitors affecting the EMT^18^.

HaCaT cells exhibit EMT as early as 36 h and more prominently at 48 or 72 h after addition of TGF-β (Fig. S1). Chemical inhibition of EMT was optimised using GW66004, a low molecular weight inhibitor of the TGF-β type I receptor kinase^19^. Incubation with GW6604 neutralised the autogenously secreted TGF-β and rendered HaCaT cultures more polarised with enhanced cell-cell adhesions. Adding GW6604 at different time points reversed TGF-β-induced EMT in HaCaT cells (Fig. S1). We adopted cell stimulations with TGF-β1 for 72 h and inhibitor addition during the last 48 h.

### High-content imaging optimisation using the TGF-β receptor kinase inhibitor

**Figure 2:**
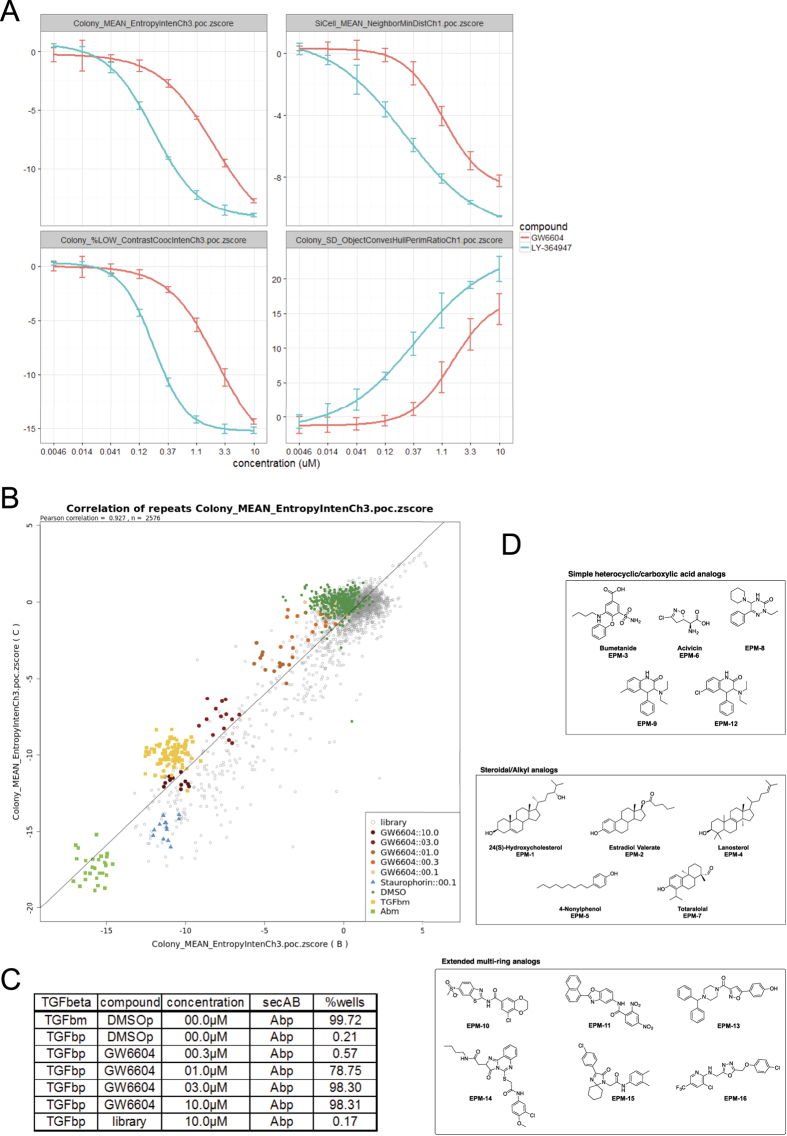
Optimisation of HCS assay using GW6604 and compound hits. (**A**) Dose-response curves of TGF-β type I receptor kinase inhibitors LY-364947 (blue curves) and GW6604 (red curves) for four different parameters. The median IC_50_ value of GW6604 was 1.9 μM and of LY-364947 was 0.2 μM. The parameters describe the texture of fibronectin signal within a colony (group of cells) (EntropyInten), the minimum distance to a neighboring nucleus (NeighborMinDist), the percentage of colonies with lower texture measurement of fibronectin signal within a colony than the threshold derived from a control population of colonies (ContrastCoocInten), and the ratio of convex hull to perimeter of a colony (ObjectConvexHullPerimeterRatio). (**B**) The scatter plot of two repeated runs of 7 plates (n = 2576) shows very good linear correlation with a Pearson coefficient of 0.927 for a single texture parameter (fibronectin signal). Negative control wells and inactive compounds scatter around the zero coordinate, whereas controls show phenotype strength-dependent scattering along the diagonal. (**C**) Table of false positive rate (FPR) and true positive rate (TPR) for the entire screen. Using the strict threshold of Euclidian distance >15 for the EMT cluster, the strong EMT inhibitor controls (GW6604: 3 and 10 μM) were identified as hits with a TPR of 98.3% and the negative control wells (DMSO) appeared as hits with a FPR of 0.21%. From the library 0.17% of wells were identified as hits. (**D**) Chemical structures of 16 compound hits. Simple heterocyclic and carboxylic acid analogs, steroidal and alkyl analogs, and extended multi-ring structures. Numbers correspond to the EPM IDs of each compound.

The assay was optimised for 96/384-well plate formats prior to the screen. In brief, several experimental conditions that also monitored sources of experimental variability, such as cell number, seeding conditions, antibody concentrations and image analysis methods were tested and analysed as a multi-factorial matrix of combinations (manuscript in preparation). Using the ‘ArrayScan’ HCS platform, HaCaT images were automatically acquired and analysed to discriminate between the epithelial (fibronectin-negative; Fig. 1B) and mesenchymal phenotype (fibronectin-positive; Fig. 1B). In addition, the six repeated experiments were analysed using two different objective magnifications and two image analysis algorithms and routines. From the entire multi-factorial data set we extracted Z′-factors calculated from cell populations exhibiting epithelial and mesenchymal phenotype. These values expressed the power by which the tested conditions for all measurements could discriminate between the two phenotypes. Finally, the most robust experimental conditions and measurements were chosen for a high-throughput screen. Two separate image analysis routines were developed to segment single cells or groups of cells as colonies. Among several hundred parameters measured, those measuring texture and relative intensity of fibronectin staining were the most reliable. A second set of parameters, nuclear density scattering and colony size-morphology were also very reliable for discriminating between epithelial (compact islets) and mesenchymal (dispersed) phenotypes (Fig. 1C). Finally, using Z′-factors and Pearson correlation coefficients from several hundred measurements, 18 parameters were selected to generate a multi-parametric profile. Among them were parameters for fibronectin, colony morphology, nuclear shape and distribution (Fig. S3A). Induction of EMT in HaCaT by TGF-β for 72 h was nearly completely reverted by co-treatment with 3.3 μM GW6604 during the last 48 h (Fig. S2A), whereas co-treatment for 24 h or 36 h only partially inhibited EMT. As a proof-of-principle, we screened a small set of 78 compounds (mostly protein kinase inhibitors) first manually in a 96-well format at 7 concentrations (41 nM–30 μM), and second, fully automated in a 384-well format at 8 concentrations (4.6 nM–10 μM) using robotic liquid handling. The proof-of-principle screens in both plate formats showed that manual and robotic screening gave similar results (unpublished results). Two independent TGF-β type I receptor kinase inhibitors appeared as hits at several concentrations (Fig. S2C); GW6004 showed effective concentrations between 1.1 and 30 μM, whereas LY-364947 showed effective concentrations between 123 nM and 10 μM. Analysis of the dose-response curves of several measurement parameters showed nice IC_50_ curves for both fibronectin texture and nuclear scattering/colony morphology (Fig. 2A). IC_50_ values of GW6604 (1.9 μM) were one order of magnitude higher than the IC_50_ values of LY-364947 (0.2 μM). Two more kinase inhibitors were identified as EMT blockers, oxindole I and glycogen synthase kinase 3β (GSK3β) inhibitor I (Fig. S2C); whereas the GSK3β inhibitor I (CAS 667463-62-9) appeared as a hit, two independent GSK3β inhibitors included in the library failed to block EMT, waiving a reliable assignment of GSK3β activity in the EMT assay. In summary, a high-content assay was developed and validated to be applicable for medium- to high-throughput screening by using robotic liquid handling, automated imaging and image analysis.

### High-throughput imaging screen for compounds that inhibit terminal EMT stages

A large screening campaign was performed in the 384-well format using the HCS platform ‘ArrayScan’. In total, 177 plates covering several compound collections with 38,302 unique compounds were screened. TGF-β type I receptor kinase inhibitors (GW6604 and LY-364947) were added as positive controls at five concentrations to generate standards of strong to very weak inhibition (Fig. S2D). In addition, staurosporine at high concentration was added to monitor toxicity. These control conditions helped data analysis, i.e. optimisation of clustering and threshold setting for hit definition. A brief flow-chart in Fig. 1D shows the data analysis process from raw data acquisition to final hit list generation. Overall, 63,897 data points were obtained from the entire screen, including control measurements and repeats of some small “focused” libraries (2–3 times) to verify repeatability of the assay. From two independent image analysis routines, 18 parameters were selected to create a multi-parametric profile for each well (Fig. S3) that described the phenotype induced by the compound. The parameters chosen were the most robust among several experiments, to a limited extent redundant and could be separated into groups of parameters describing fibronectin expression, size and morphology of colonies, distribution of nuclei, intensity and shape of nuclei and performance of the assay. Using multi-parametric profiles, toxic and EMT phenotypes were easily discriminated and the phenotypic strength of positive controls at different compound concentrations was very well represented (Fig. S3).

Since most of the compounds were screened as a single data point, the reproducibility of the assay was analysed using repeats of a small “focused” library of 2,000 compounds. The Pearson correlation coefficient for a single measurement parameter was 0.927 (Fig. 2B), indicating a very high reproducibility. The overall correlation for all 18 parameters between three independent repeats was around 0.8 (Fig. S4A) showing that all chosen parameters were very reproducible. Next, the quality of the assay was measured by calculation of the Z′-factor^17^, a statistical parameter often used in the field of high-throughput screening. For the TGF-β receptor controls and the toxicity controls, the median Z′-factors calculated from the Euclidian distance of the normalised multi-parametric profiles indicated that the assay was excellent for screening (GW6604 10 μM, Z′ = 0.69; staurosporine 0.1 μM, Z′ = 0.66) (Fig. S4B). After normalisation of the data^20^, clustering and threshold-based filtering, 93 unique compounds were identified as hits (Fig. 1D). The data analysis was done using the open-source data mining software KNIME^21^, and all statistical methods including normalisation, clustering, and threshold-based filtering were carefully optimised using the positive and negative controls. Finally, based on the analysis of the entire screen, the true positive rate (TPR) was 98.3% for the EMT inhibitor controls (GW6604, 10 and 3 μM) and the false positive rate (FPR) was 0.21% for the dimethyl-sulfoxide (DMSO)-treated control cells. There were two minor technical issues in the vehicle controls which increased the FPR; after excluding affected wells the FPR was 0.02%. The FPR of the toxicity control was 0%, and the hit rate of the library samples varied depending on the compound collection between 1.88% for a small focused library of kinase inhibitors to ~0.12% for compound collections with unknown biological activity. The overall hit rate for the library was 0.28%; without taking two focused libraries into account the hit rate was 0.17% (Fig. 2C). This demonstrates that the primary screen was highly specific and reproducible.

The hits were then verified and validated using a secondary assay measuring the effect on the direct downstream substrate of the TGF-β type I receptor, Smad2, which is phosphorylated at its C-terminus (p-Smad2) upon TGF-β signalling and translocates into the nucleus. p-Smad2 is a good marker for early TGF-β signalling and could be used to discriminate between general TGF-β inhibitors and selective late-stage EMT inhibitors (Fig. S4C). Translocation of p-Smad2 occurred within tens of minutes and the optimal time point of the assay determined via time course experiments was 1 hour after stimulation (Fig. S4D), which is in line with kinetic models obtained from experimental data in HaCaT cells^22^. Hits having no or little effect on translocation of p-Smad2 were selected as the most interesting compounds (Fig. S5). In addition, compounds that could not be verified and failed to generate reproducible phenotypes were also excluded. In summary, 35 compounds (38% of all hits) gave once or twice a reproducible EMT phenotype in two experimental repeats (Figs S5 and S6A). Scatter plot analysis of p-Smad2 nuclear translocation versus strength of phenotype allowed us divide the 35 verified compounds into groups of compounds that behaved like TGF-β receptor inhibitors and compounds that had no or little effect on p-Smad2 nuclear translocation (Fig. S6B). We found three compounds (CBN::49::1::14, CBN::42::7::9 and KBI::2::5::6) that inhibited p-Smad2 nuclear translocation and which had similarities in their molecular structure with known TGF-β receptor inhibitors (Fig. S6C). In fact, KBI::2::5::6 is an established TGF-β type I receptor inhibitor and is widely known as SB-431542. This is in agreement with independent high content screens for EMT inhibitors, where the majority of chemical compounds identified are proven to act as TGF-β receptor inhibitors^18^. Considering all experimental data points, the molecular structure of the hits and certain proprietary reasons, 19 compounds were finally excluded from the hit list (Figs S5 and S6A,B). However, deeper analysis of the excluded compounds may be interesting in terms of uncovering novel modulators of TGF-β/Smad signaling.

After analysis of the verification and the counter-screen, 16 compounds were identified as candidates for inhibition of EMT without affecting TGF-β-induced p-Smad2 nuclear accumulation (Fig. 1D). The hits could be classified into three chemical classes: simple heterocyclic and carboxylic acid analogs (5 compounds), steroidal-alkyl analogs (5 compounds) and extended multi-ring analogs (6 compounds) (Fig. 2D). We conclude that the high-content screen identified specific chemical compounds that block terminal EMT and mesenchymal-specific fibronectin accumulation without affecting basal TGF-β signalling. None of the identified compounds resembled chemicals with previously described functional roles in modulating epithelial plasticity.

### A selective sub-group of compounds points to the action of hydroxycholesterol

**Figure 3:**
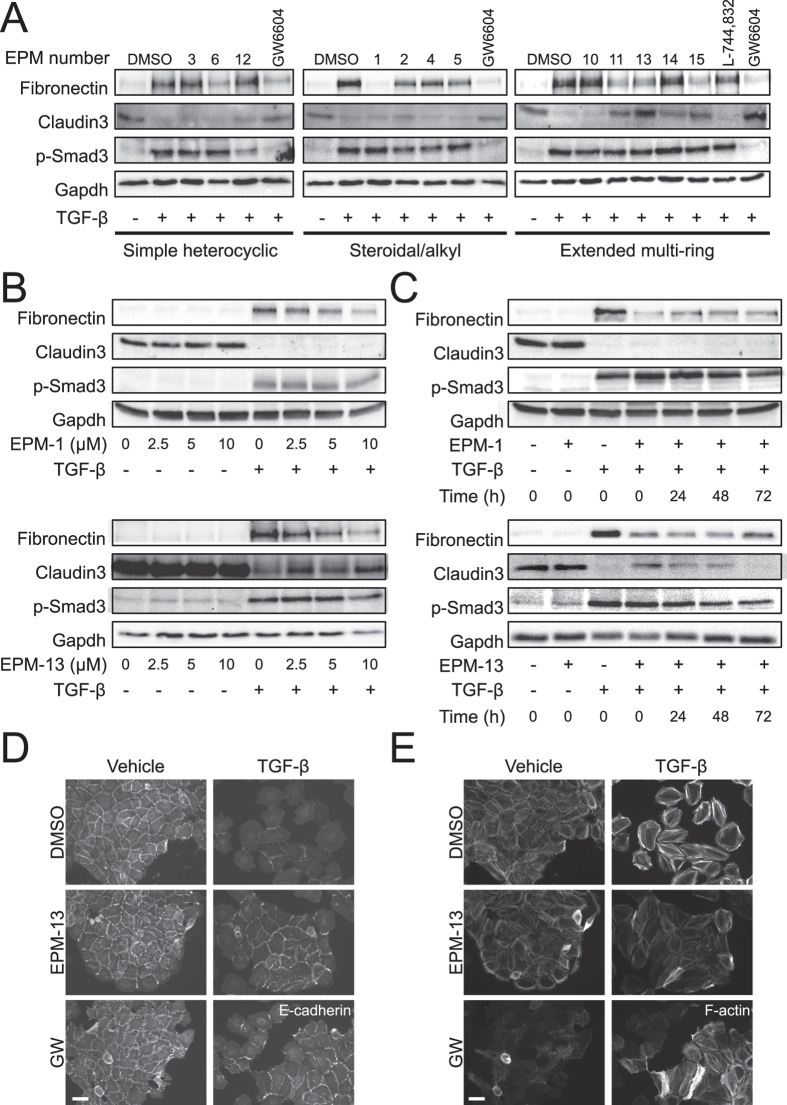
Analysis of 13 selected compounds in keratinocytes. (**A**–**C**) Protein expression analysis in HaCaT total cell lysates stimulated (+) or not (−) with 5 ng/ml TGF-β for 96 h. In (**A**) cells were co-treated with DMSO or specific EPMs (10 μM). In (**B**) cells were co-treated with DMSO (0 μM) or EPM-1 and EPM-13 at the indicated concentrations. In (**C**) cells were co-treated with DMSO (0 μM) or EPM-1 and EPM-13 (10 μM) at the indicated time points following TGF-β stimulation. Immunoblots for the indicated proteins and Gapdh, the protein loading control, are shown. (**D**) E-cadherin immunofluorescence microscopy of HaCaT cells stimulated with vehicle or 5 ng/ml TGF-β for 96 h in the presence of DMSO or 10 μM of EPM-13 or 3.3 μM GW6604 (GW). (**E**) Actin microfilament direct fluorescence microscopy of HaCaT cells treated as in panel D. White bars indicate 10 μm.

Based on their commercial availability, we focused on 13 of the 16 confirmed hits from the screen (Fig. 2D). We refer to these compounds as epithelial plasticity modulators (EPMs) and in the case where the compounds have known biological effects, their common names are also listed. We verified the activities of these hits in HaCaT cells by analysing their effects (from low nM to a maximum of 10 μM) on epithelial-mesenchymal protein markers, but for simplicity we present selected sets of expression data i.e. on the mesenchymal fibronectin, the epithelial claudin-3 and Smad3 C-terminal phosphorylation (p-Smad3) as a direct readout of TGF-β signalling (Fig. 3A). Inhibitor GW6604 served as a positive control; it inhibited phosphorylation of Smad3, blocked fibronectin and restored claudin-3 expression. The 13 hits demonstrated distinct phenotypic profiles with respect to these three markers (see also summary in Table S1). For example, EPM-6 (acivicin) partially blocked TGF-β-induced fibronectin expression without reverting the claudin-3 levels, and left p-Smad3 intact (Fig. 3A). EPM-12 showed a partial reversion of claudin-3 without affecting strongly fibronectin, however, this compound weakly decreased p-Smad3 (Fig. 3A). EPM-1 (24(S)-hydroxycholesterol), EPM-2 (estradiol valerate), EPM-4 (lanosterol), and EPM-5 (4-nonylphenol), all had inhibitory effects on fibronectin without perturbing claudin-3 or p-Smad3 (Fig. 3A). Among the extended multi-ring compounds, EPM-11, 13 and 15 had the strongest effects, and inhibited not only fibronectin but also reverted the epithelial claudin-3, again without showing significant interference with p-Smad3 activation (Fig. 3A). Based on their potency and effects, a few compounds were selected for further studies.

EPM-1 dose-dependently blocked fibronectin expression without affecting epithelial claudin-3 (Fig. 3B). EPM-1 was a more potent inhibitor when added simultaneously with TGF-β1, but its inhibitory activity persisted even when added 72 h after TGF-β1 stimulation (Fig. 3C). EPM-13 exhibited a similar potency to EPM-1 in blocking fibronectin, but also partially reverted epithelial claudin-3 levels (Fig. 3B) and seemed to have the strongest effect when added simultaneously with TGF-β (Fig. 3C). The action of EPM-13 as an anti-EMT blocker was verified by immunofluorescence experiments; in the presence of EPM-13, TGF-β-induced mesenchymal cells regained their plasma membrane E-cadherin and downregulated their intense stress fibers, exhibiting a morphological reversion comparable to the reversion caused by GW6604 (Fig. 3D,E). A similar result was found for EPM-10, a second extended multi-ring inhibitor; although EPM-10 did not score well for reversion of the epithelial claudin-3 or CAR upon immunoblot analysis (Fig. S7A,B); immunofluorescence microscopy clearly verified that EPM-10 blocked the pro-EMT effect of TGF-β and generated tightly assembled cell islets with well formed adherens junctions and cortical actin (Fig. S7C–E). Using the HaCaT islet density assay described for the high-content screen, we also verified that both EPM-10 and EPM-13 induced reappearance of dense islets, similar to the effect of GW6604, when compared to the intense cell scattering induced by TGF-β (Figs S7E and S8A,B).

**Figure 4:**
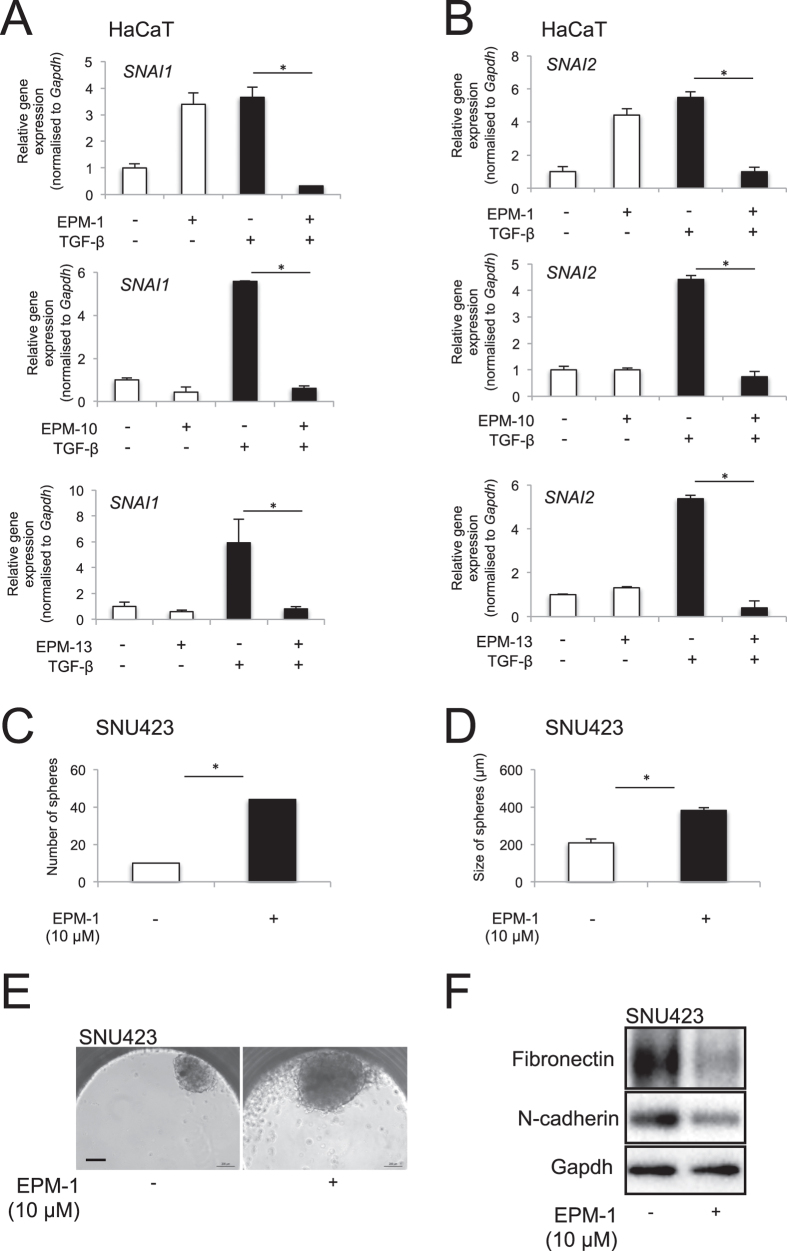
Compounds that could suppress EMT transcription factor expression in HaCaT cells. (**A**,**B**) mRNA expression analysis of *Snail1* (**A**) and *Snail2* (**B**) in HaCaT cells in the absence or presence of 5 ng/ml TGF-β for 72 h, and in the presence of DMSO or specific compound (EPM-1, EPM-10 and EPM-13), analysed by real-time RT-PCR and normalised against the housekeeping *Gapdh* mRNA. The data are expressed as bar graphs of average determinations with corresponding standard errors from triplicate determinations. Stars indicate significant difference at *p* < 0.05. (**C**,**D**) Number (**C**) and size (**D**) of hepatospheres grown in hanging drops using Insphero assays in the presence of control, DMSO or 10 μM EPM-1. The data are expressed as bar graphs of average determinations with corresponding standard errors from triplicate determinations. Stars indicate significant difference at *p* < 0.05. (**E**) Representative phase contrast images of hepatospheres grown in hanging drops using Insphero assays in the presence of control, DMSO or 10 μM EPM-1. (**F**) Protein expression analysis in the hepatospheres treated with DMSO or 10 μM EPM-1 under the same conditions as in panels C-E. Immunoblots for the indicated proteins and Gapdh, the protein loading control, are shown.

Since EMT is mediated by a specific group of transcription factors and chromatin regulators^15^,^16^, we assayed the expression of several members of this functional group that are potently induced by TGF-β in HaCaT cells, including Snail1, Snail2, ZEB1, Twist1 and high mobility group A2 (HMGA2) (Fig. 4A,B). As expected, EPM-10 and EPM-13 blocked the induction of Snail1 and Snail2 by TGF-β in HaCaT cells, but even EPM-1 potently suppressed Snail1 and Snail2 induction by TGF-β (Fig. 4A,B). Unexpectedly though, EPM-1 seemed to reproducibly induce Snail1 and Snail2 expression in the absence of TGF-β stimulation, an observation that could not be correlated with the basal effects of EPM-1 on epithelial and mesenchymal markers in the same cells (Fig. 3). In contrast EPM-10 and EPM-13 had no effect on basal Snail1 or Snail2 expression (Fig. 4A,B). On the other hand, ZEB1, Twist1 and HMGA2 expression did not significantly change in the presence of these compounds in HaCaT cells (unpublished results). In a two-dimensional migration assay of human prostatic carcinoma PC3U cells, after a linear wound, EPM-13 failed to block cell migration, whereas EPM-10 was as potent as GW6604 (Fig. S8C). Collectively, the various cell-based assays confirmed that individual EPMs exhibited distinct profiles of biological activities.

We confirmed compound bioactivity in human cancer cells by testing all 13 compounds in lung adenocarcinoma A549 (not shown), breast cancer MCF10A-MII (Fig. S9) and hepatocellular carcinoma (HCC) Hep3B cells (Fig. S10); the results were comparable to HaCaT cells. In the MCF10A-MII cell model, the impact of many compounds was evident by microscopic analysis of general cell morphology (Fig. S9A), downregulation of fibronectin expression without corresponding effects on E-cadherin or plasminogen activator inhibitor 1 (PAI-1), the latter serving as a marker of TGF-β responsiveness that is independent of cell type (Fig. S9B). In HCCs, EPM-5, -6, -11 and -12 reproducibly blocked the expression of fibronectin, without strong effects on E-cadherin expression (Fig. S10A–C).

We also tested whether specific compounds could affect cancer stem cell features known to be functionally linked to the process of EMT^1^, or whether the compounds could alter the mesenchymal phenotype of tumor cells outside the context of TGF-β signaling. Experiments were performed in the mesenchymal HCC cell model SNU423, for which 3-dimensional hepatospheres can be generated by culturing the cells using the hanging drop method (Figs 4C–F and S11). In these assays, mainly EPM-1, EPM-10 and EPM-13 were analysed. Whereas EPM-1 had a significant effect on increasing the number and size of spheres (Fig. 4C–E), EPM-10 and EPM-13 did not significantly alter the hepatosphere phenotype, although a trend towards increased sphere size could be recorded (Fig. S11). As these results were not encouraging, we did not perform a deeper analysis of the effects of these compounds on the expression of marker genes for cancer stem cells. However, analysis of mesenchymal proteins such as fibronectin and N-cadherin that are constitutively expressed by SNU423 cells were found to be significantly downregulated by EPM-1 and EPM-10 in the hepatospheres (Figs 4F and S11D), whereas EPM-13 had no clear effect on these two mesenchymal proteins (Fig. S11H). It is therefore possible that some of these compounds could suppress mesenchymal cell features of tumor cells but further analysis will be required.

Based on the various cell models employed thus far in the study, we conclude that select compounds identified using our screening approach reverted mesenchymal cells back to the epithelial phenotype (EPM-10/-13), whereas the majority of compounds, including EPM-1, showed good inhibition of mesenchymal features without fully reverting cells to an epithelial phenotype. A summary of the activity of the tested compounds appears in Table S1.

### Inhibition of myofibroblast differentiation

**Figure 5:**
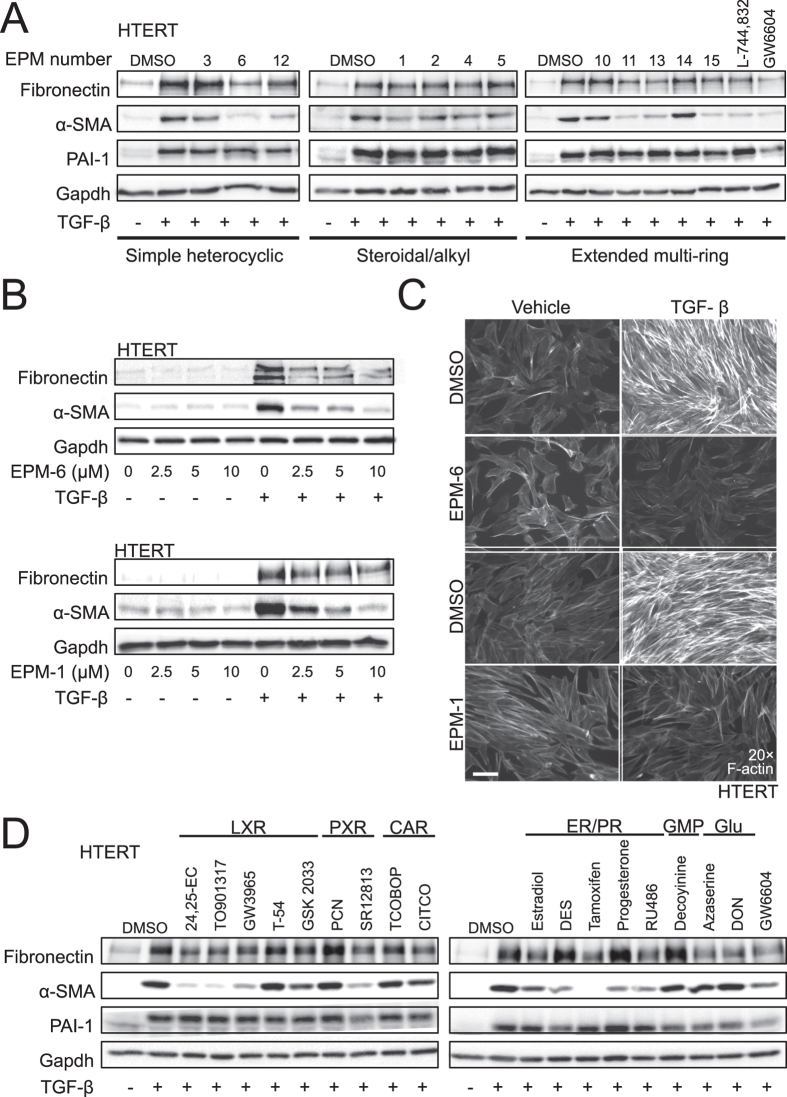
Analysis of 13 selected compounds in HTERT fibroblasts. (**A**,**B**) Protein expression analysis in HTERT total cell lysates stimulated (+) or not (−) with 5 ng/ml TGF-β for 72 h and co-treated with (**A**) DMSO or specific EPMs (10 μM); (**B**) DMSO (0 μM) or EPM-6 and EPM-1 at the indicated concentrations. Immunoblots for the indicated proteins and for Gapdh, the protein loading control, are shown. (**C**) Actin microfilament direct fluorescence microscopy of HTERT cells stimulated with vehicle or 5 ng/ml TGF-β for 72 h in the presence of DMSO or 10 μM of EPM-6 and EPM-1. White bar indicates 10 μm. (**D**) Protein expression analysis in HTERT total cell lysates stimulated (+) or not (−) with 5 ng/ml TGF-β for 72 h and co-treated with DMSO or specific compounds (10 μM). Immunoblot for the indicated proteins and for Gapdh, the protein loading control, are shown.

EMT generates transitory mesenchymal cells, and resembles the TGF-β-induced conversion of fibroblasts to terminally differentiated myofibroblasts^8^. Myofibroblasts specialise in matrix production, and our lead compounds inhibited expression of the matrix component fibronectin in response to TGF-β. We therefore hypothesised that these compounds might block the matrix-producing myofibroblast phenotype. We tested all 13 compounds for their effects in a model of myofibroblast differentiation using human immortalised fibroblasts HTERT (Fig. 5). αSMA was monitored as a specific marker of myofibroblast differentiation, and PAI-1 as a marker of TGF-β responsiveness (Fig. 5A). The compounds blocked fibronectin expression without affecting TGF-β-induced PAI-1 expression; however, EPM-1, -2, -4, -6, -11, -12, -13 and -15, strongly blocked αSMA expression and did so more potently than inhibiting fibronectin (Fig. 5A). The effects were confirmed in dose-dependent experiments (Fig. 5B showing EPM-6 and -1). The quantitative effect on protein expression was also evident at the level of total actin microfilaments stained with phalloidin (Fig. 5C). TGF-β induced a strong F-actin network that was blocked by EPM-6 and EPM-1.

Based on their reported biological mechanisms, we expanded our characterisation of EPM-1, -2, -5 and -6 by testing analogs of these compounds that have known cellular targets. EPM-6 (acivicin) is an irreversible inhibitor of glutamine-dependent amidotransferases including glutamate synthase and GMP synthase (as well as enzymes such as γ-glutamyl transpeptidase), and has been tested as a chemotherapeutic against various human cancers^23^,^24^. In order to evaluate the impact of broadly inhibiting glutamine-based metabolism and specific enzymes targeted by acivicin^25^, we tested the glutamine analogs azaserine and DON^23^, and the GMP synthase inhibitor decoyinine. None of these compounds had any impact on αSMA, but the two glutamine analogs, azaserine and DON, significantly decreased fibronectin expression (Fig. 5D). Hence, it is likely that acivicin affects cell plasticity not via effects on glutamine metabolism *per se* but via as yet unknown bioactivities.

Intriguingly, EPM-1 (24(S)-hydroxycholesterol), EPM-2 (estradiol valerate) and EPM-5 (4-nonylphenol) are known agonists of the liver X receptors, the estrogen receptors (ERs), and the pregnane X receptor (PXR), respectively. Given that our screening strategy identified multiple nuclear receptor ligands, we tested an expanded set of nuclear receptor agonists and antagonists to identify potential targets with the greatest impact on myofibroblast differentiation. This list of compounds included agonists and antagonists of the LXRs, ERs, progesterone receptor (PR), PXR and constitutive androstane receptor (CAnR) (Fig. S12A). Like EPM-1, a natural (24(S), 25-epoxycholesterol) and two synthetic (T0901317, GW3965) LXR agonists potently blocked αSMA and fibronectin induction by TGF-β in HTERT fibroblasts, whereas two LXR antagonists (Tularik Compound 54, GSK 2033) had no effect (Fig. 5D). No obvious effect on general TGF-β signalling was observed as assessed by monitoring PAI-1 expression (Fig. 5D). The PXR agonist SR12813 blocked αSMA levels but also affected PAI-1, whereas another, pregnenolone-16α-carbonitrile (PCN), had no effects. The two CAnR agonists, TCPOBOP and CITCO, had weak effects. Finally, the ER and PR ligands showed equally potent inhibitory effects against αSMA, with tamoxifen exhibiting most potent inhibition against αSMA expression but variable effects on fibronectin levels (Fig. 5D).

All these compounds were also tested in the HCC Hep3B model in addition to a few more compounds, including two synthetic LXR agonists (GSK3987, WYE672), which downregulated TGF-β-induced fibronectin (Fig. S10 and unpublished results). Further, consistent with their lack of activity on the LXRs, 24(R)-27-hydroxycholesterol and 22(R)-hydroxycholesterol had no impact on fibronectin in Hep3B cells (Fig. S10A). Overall, similar to fibroblasts or other epithelial cells, all LXR agonists reduced HCC fibronectin expression in a dose-dependent manner (Fig. S10D,E).

**Figure 6:**
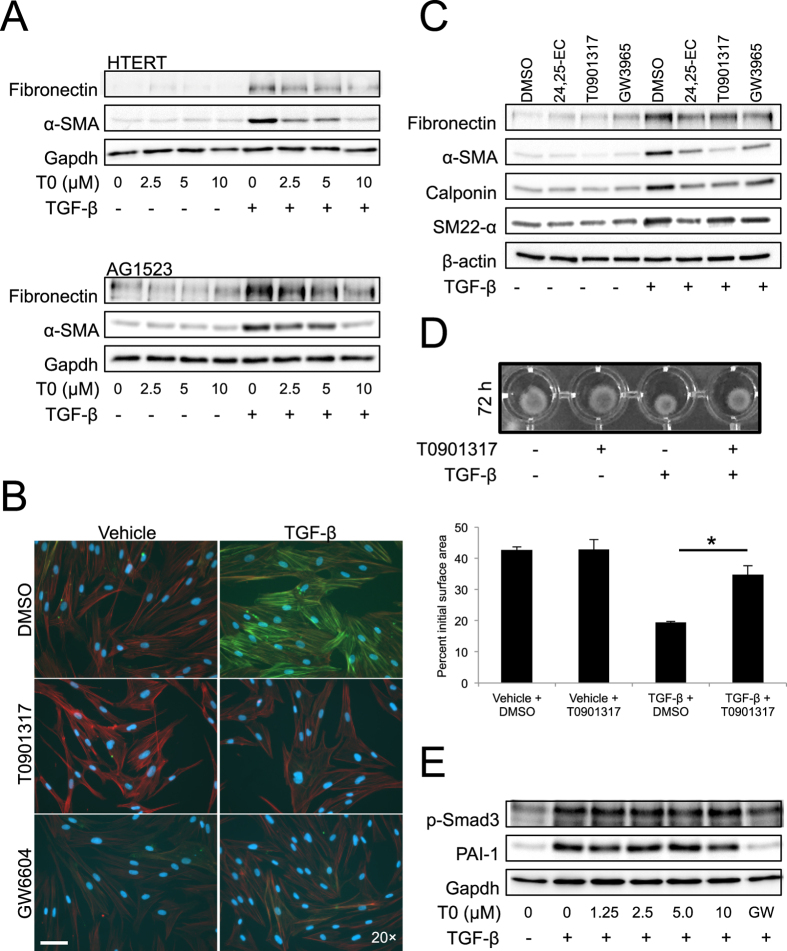
LXR agonists block myofibroblast differentiation. (**A**) Protein expression analysis in HTERT and AG1523 total cell lysates stimulated (+) or not (−) with 5 ng/ml TGF-β for 72 h and co-treated with DMSO (0 μM) or specific T0901317 (T0) compound at the indicated concentrations. Immunoblots for the indicated proteins and for Gapdh, the protein loading control, are shown. (**B**) αSMA microfilament immunofluorescence microscopy in AG1523 cells stimulated with vehicle or 5 ng/ml TGF-β for 72 h in the presence of DMSO or 10 μM of T0901317 and 3.3 μM GW6604 (GW). Images stained blue for DAPI-positive nuclei, red for F-actin microfilaments and green for αSMA microfilaments. White bar indicates 10 μm. (**C**) Protein expression analysis in AG1523 total cell lysates stimulated (+) or not (−) with 5 ng/ml TGF-β for 72 h in the presence of DMSO (control) or the indicated LXR agonists. β-Actin serves as a loading control. (**D**) Collagen gel contraction assay performed on AG1523 cells stimulated (+) or not (−) with 5 ng/ml TGF-β for 72 h in the presence of LXR agonist T0901317 or DMSO (control). A representative image and corresponding quantification of contracted gels graphed as average of 5 repeats with associated standard deviation. A star indicates statistically significant difference at *p* < 0.05. (**E**) Protein expression analysis in AG1523 total cell lysates stimulated (+) or not (−) with 5 ng/ml TGF-β for 24 h and co-treated with DMSO (0 μM) or specific T0901317 (T0) compound at the indicated concentrations. Immunoblots for the indicated proteins and for Gapdh, the protein loading control, are shown.

Based on the robust pharmacology of LXR ligands observed in our screening, we focused on understanding their impact on myofibroblast differentiation. In two different human fibroblast models that differentiate to myofibroblasts in response to TGF-β (HTERT and primary AG1523), all LXR agonists tested dose-dependently blocked TGF-β-inducible fibronectin and αSMA (Fig. 6A,B showing data for T0901317; Fig. S12C–E showing data for 24, 25-EC and GW3965). Microscopic analysis also demonstrated a block of actin microfilament assemblies by all three LXR agonists and most potently by GW3965 (Fig. S12E). Side-by-side comparison of each of these agonists showed a strong reduction in the TGF-β-induced expression of additional myofibroblast markers (calponin, SM22-α) without affecting basal levels of the proteins (Fig. 6C). Functionally, T0901317 inhibited TGF-β-mediated contraction of collagen gels by differentiated myofibroblasts (Fig. 6D, 19.5 ± 0.3% versus 34.8 ± 2.8% of initial gel area, p < 0.05). Results were confirmed using additional LXR agonists (24, 25-EC and GW3965) or the LXR antagonist GSK2033 (Fig. S13). The LXR agonist, 24, 25-EC, which is known to be less potent, blocked TGF-β-induced collagen contraction but not as efficiently as the other two agonists (Fig. S13A). The GW3965 agonist also blocked TGF-β-induced collagen gel contraction (Fig. S13B, reproducing the effect of T0901317 (Fig. 6D and Fig. S13C showing data at 48 h). Finally, the LXR antagonist, GSK2033, had no impact on TGF-β-induced collagen gel contraction (Fig. S13D). As previously stated, T0901317 did not reduce central TGF-β signalling (monitored as p-Smad3 and PAI-1, Fig. 6E). We conclude that agonistic activation of LXR function blocks myofibroblast differentiation induced by TGF-β.

### LXRα protects fibroblasts from myofibroblast differentiation

**Figure 7:**
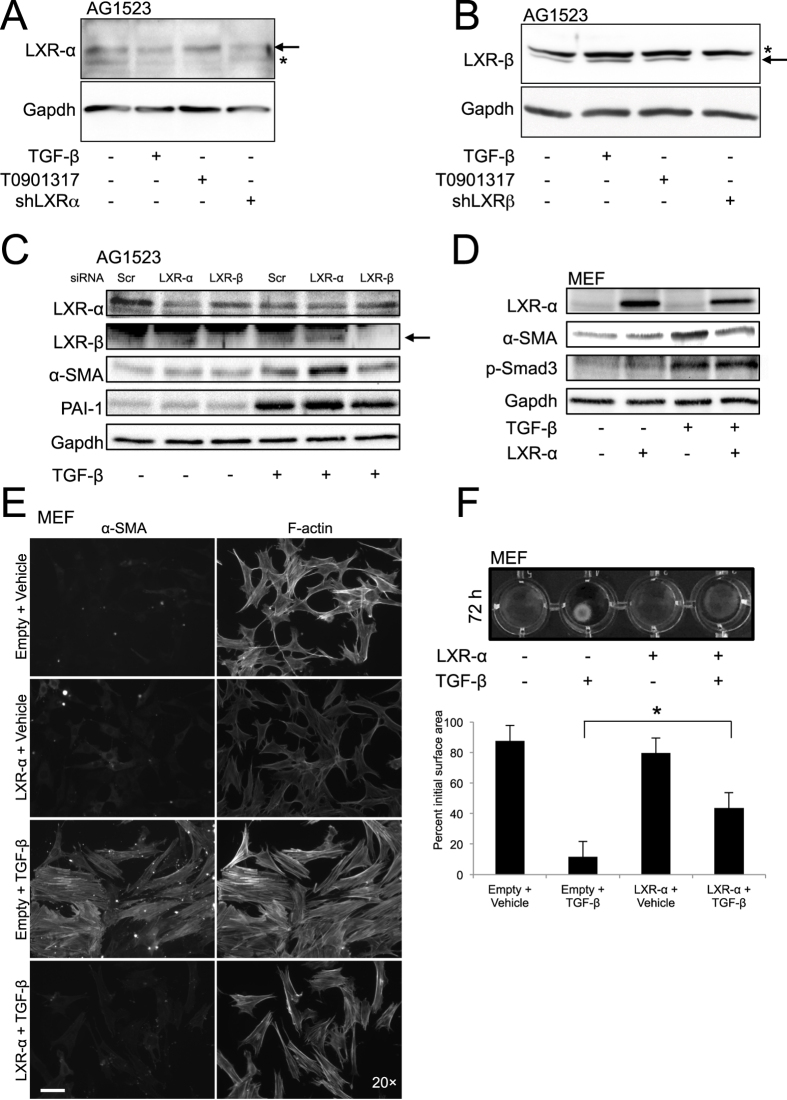
LXRα inhibits TGF-β-induced myofibroblast differentiation. (**A**–**D**) Protein expression analysis in AG1523 (**A**–**C**) and MEF (**D**) total cell lysates stimulated (+) or not (−) with 5 ng/ml TGF-β for 72 h (**A**–**D**) or 24 h (**C**). In (**A**,**B**) AG1523 cells were stimulated with the LXR agonist T0901317 for 24 h in order to stabilise LXRα and LXRβ, thus providing evidence for the specificity of the detected protein band. In (A, B) AG1523 cells were also transfected with shRNA vectors targeting LXRα (**A**) and LXRβ (**B**) in order to show specificity of the detected protein band. In (**C**) AG1523 were transfected with the indicated siRNAs. In (**D**) MEFs were transiently transfected with LXRα cDNA vector. Immunoblots for the indicated proteins and for Gapdh, the protein loading control, are shown. Arrows mark the specific protein bands. (**E**) Direct and immunofluorescence microscopy of MEFs transfected and stimulated as in panel C and analysed for total F-actin and αSMA microfilaments. White bar indicates 10 μm. (**F**) Collagen gel contraction assay of MEFs transfected with LXRα cDNA vector (or control vector) and stimulated (+) or not (−) with 5 ng/ml TGF-β for 72 h. Quantification of the surface area of contracted gels is presented as in Fig. 6D (star: statistically significant difference at *p* < 0.05).

As the previous experiments analysed physiological effects of the LXR agonists on fibroblasts (Fig. 6), we next assayed for LXR expression in these cells (Figs 7A and S14A,B). Detectable mRNA levels of both *LXRα* and *LXRβ* were measured in AG1523 fibroblasts (Fig. S14A). Interestingly, TGF-β stimulation suppressed *LXRα* expression (>50%), whereas *LXRβ* was induced up to 1.5-fold (Fig. S14A). The expression and regulation of LXR isoforms by TGF-β was confirmed at the protein level (Figs 7A and S14B). Since the LXR proteins are expressed at rather low levels in the cells that we analysed, and available antibodies are not so reliable, we confirmed the identity of each LXR isoform after transfection of the fibroblasts with short hairpin RNAs that target each specific isoform (Fig. 7AB). Whereas T0901317 reproducibly stabilised the protein levels of LXRα and LXRβ, TGF-β downregulated LXRα and upregulated LXRβ to a low but significant degree (Figs 7AB, S14B). In order to explore the roles of LXRα and LXRβ in myofibroblast differentiation, transient knockdown of LXRα or LXRβ was effective at both mRNA and protein levels (Figs 7C and S14C). TGF-β-induced αSMA expression was further enhanced after silencing LXRα, whereas LXRβ silencing had no effect (Figs 7C and S14C). The LXRα knockdown was not sufficient to increase αSMA in the absence of TGF-β and this is most likely because in this cell system, myofibroblast differentiation is TGF-β-dependent. Since LXRα knockdown does not activate autocrine TGF-β signaling, the differentiation process remains dependent on exposure to exogenous TGF-β. Conversely, transient expression of exogenous LXRα in mouse embryonic fibroblasts (MEFs) caused reproducible repression of TGF-β-induced αSMA (Fig. 7D). Fluorescence microscopy for endogenous F-actin and αSMA demonstrated that LXRα downregulated completely αSMA and to a lower extent F-actin microfilaments (Fig. 7E). Silencing or overexpression of LXRα had no effect on p-Smad3 or PAI-1 activation by TGF-β (Fig. 7C,D), neither did we observe effects of the LXR agonist (T0191317) on the activity of a Smad-specific promoter-luciferase reporter (CAGA_12_-luc) (Fig. S14D), nor did silencing of endogenous LXRα affect the activity of the CAGA_12_-luc reporter (unpublished results). Functionally, LXRα overexpression inhibited TGF-β-mediated contraction of collagen gels by myofibroblasts (Fig. 7F, 11.7±0.9% versus 43.6±3.4% of initial gel area, p < 0.05). Collectively, these data suggest that TGF-β negatively regulates LXRα in fibroblasts, which is physiologically important as LXRα counteracts the pro-fibrotic effect of TGF-β.

## Discussion

Our screen for chemical modulators of EMT identified compounds with diverse biological activities and included new compounds that affect myofibroblast differentiation, a process relevant to fibrosis and cancer^7^,^8^,^11^. The relatively small number (16) of final hits identified show structural diversity, providing a diverse group of compounds that affect epithelial plasticity and myofibroblast differentiation in distinct ways (Table S1). These compounds may find use in further studies of the intermediate stages of EMT, the deeper understanding of myofibroblast differentiation and, as we discuss later for LXR agonists, they may also suggest new ways of combinatorial treatment for fibrotic and cancer pathologies.

Because the analysis of epithelial plasticity largely depends on cell biological parameters^3^,^4^,^26^, cell-based screens using high-content imaging systems are important^27^,^28^. This technology has advanced dramatically, and has been applied to cell biological processes as complex as vesicular trafficking^29^,^30^.

The HaCaT keratinocytes employed are well studied in the TGF-β field as they faithfully undergo EMT^14^,^15^,^16^. Due to the discriminative power of the high-content robotic microscope, we used a single marker of mesenchymal/fibroblast differentiation, i.e. fibronectin, which was sufficient to generate successful chemical hits from the screen. Although our initial plan was to use multiple markers in order to more accurately monitor the reversion of EMT, analysis of the expression and localisation of markers such as E-cadherin, vimentin and the actin cytoskeleton (unpublished results and Fig. S2B), did not generate reliable discriminatory power or reproducible imaging profiles that were sufficient for quantitative analysis in a high-throughput setting. This experience coincides with findings from the recently published imaging screen that also scored fibronectin as a reliable marker of “terminal” stages of EMT, which could then be reverted back to its basal epithelial levels^18^. Despite the current limitations, we anticipate that future screens based on multiple protein markers may allow for a more complex analysis of intermediate stages and sub-programs of the EMT process. Although the cell model employed was absolutely dependent on stimulations with exogenous TGF-β, the counter-screen against direct regulators of Smad phosphorylation coupled with the late time point of compound addition allowed us to study the late stages of plasticity progression; we propose that late stages depend on initial TGF-β stimulation but are eventually sustained by other factors.

The detailed evaluation of the 13 compounds provided some general points. Certain extended multi-ring scaffolds can potently revert a mesenchymal phenotype towards an epithelial (EPM-10 and -13, Figs 3 and 4 and S7–9). EPM-10 and -13 promote the rebuilding of adherens junctions without blocking TGF-β signalling or rescuing the temporal degradation of tight junction components (Figs 3 and S7). Searches based on the molecular structure of these compounds have not yet allowed us to identify specific cellular targets of their activity. An unbiased approach of molecularly “fishing” for biomolecules that associate with EPM-10/-13 may provide interesting clues for their targets. In addition to these compounds, acivicin, an inhibitor of glutamate, nucleoside and glutathione biosynthesis, clearly regulates epithelial plasticity (Figs 3 and 4). However, the lack of similar effects by azaserine and DON raises doubts about the general importance of glutamine metabolism on plasticity and point to other, unknown targets of acivicin. Acivicin has been extensively studied in the context of cancer, but failed in several clinical trials due to toxicity issues^23^,^24^.

Several groups of nuclear receptor agonists scored prominently in our screens, such as the LXR and ER/PR ligands (Figs 5D, 6 and S10). While the findings with ER/PR ligands are interesting, we focused on LXR because of the robust and consistent results seen with all compounds tested from this family (Fig. 5). Clearly, LXR agonism inhibits progression of fibroblasts to the myofibroblast phenotype or epithelial HCC to the mesenchymal phenotype (Figs 6 and S12 and S13). A good area for deeper elucidation of the crosstalk between LXR and TGF-β signalling is the *αSMA* gene and its regulatory sequences. Our mechanistic evidence shows a negative role of LXRα on TGF-β-mediated myofibroblast differentiation and *αSMA* gene regulation (Figs 7 and S14). In addition, TGF-β signalling negatively regulates the abundance of LXRα in fibroblasts (Figs 7A and S14A,B), suggesting that the fibrotic program of TGF-β may depend on removal of the protective role of LXRα. Since the biological functions of LXRs are well understood in the liver, the role of LXR in modulating liver fibrosis and cancer progression is worth examining in detail. Observations in mouse models provide further support for this notion. LXRα (but not LXRβ) knockout mice exhibited stromal overgrowth around the prostatic epithelium, with high levels of αSMA and high TGF-β signalling activity in the stroma^31^. Furthermore, the LXR agonist T0901317 was shown to protect against bleomycin-induced mouse skin fibrosis; however, LXR function in this model was found to be directed towards resident macrophages that secreted interleukin-6, which then suppressed myofibroblast differentiation^32^. Recently, in a model of cardiac fibrosis after aortic constriction, the LXR agonist AZ876 successfully blocked fibrosis by inhibiting the effects of TGF-β on vascular smooth muscle differentiation and αSMA induction^33^. Although some of these *in vivo* studies favor the action of macrophages or other unidentified stromal cells as those that respond to LXR agonists^31^,^33^, our evidence using three established fibroblast models (HTERT, AG1523 and MEFs) suggests that fibroblasts can respond to LXR agonists and even more importantly, LXR levels can be modulated by TGF-β signalling (Figs 7 and S14). Overall, the potential clinical utility of LXR agonists for lipid disorders^12^,^13^, and our evidence from *in vitro* cell models, suggest that LXR agonists may be useful for future combinatorial treatments of tissue fibrosis and advanced stages of cancer.

Functions of different members of the nuclear receptor superfamily have been previously linked to the regulation of the EMT in a diverse set of cancer and fibrotic conditions^34^,^35^,^36^. The estrogen receptor α (ERα) protects epithelial differentiation and acts as an anti-EMT factor especially in the mammary gland, as it directly represses the expression of Snail2; conversely, Snail1 transcriptionally represses the *ERα* gene during EMT^34^. Similar functions have been etsbalished for ERβ in the prostate, where loss of ERβ promotes Snail1 function by releasing expression of the vascular endothelial growth factor α^37^. The glucocorticoid and androgen receptors also block EMT progression, although the case of the androgen receptor in the prostate is complicated as both positive and negative effects of this protein on EMT have been reported^36^. The peroxisome proliferator-activated receptor γ (PPARγ) and its agonists block TGF-β-induced EMT in lung cancer cells and suppress metastasis in recipient mice^38^. PPARγ seems to act as a transcriptional repressor of nuclear Smad3 activity, thus limiting the potential of TGF-β to regulate the target genes that enforce the EMT. On the other hand, the retinoic acid receptor β contributes to the generation of a myofibroblast-rich stroma that promotes breast cancer progression^39^.

Nuclear receptor function, in particular PPARγ, has also been linked to tissue fibrosis by regulating both EMT and myofibroblast activation^40^,^41^. Many agonists of PPARγ have shown promising anti-fibrotic effects in the lung and other organs^40^,^41^, and the activity of PPARγ is important in epithelial cells but also in stromal fibroblasts, where their activation towards myofibroblasts is blocked by PPARγ agonists. However, the action of PPARγ agonists may involve more complex mechanisms, even independent from this receptor^40^,^41^.

Systematic screens for agents that affect plastic changes in epithelial tissues have shown utility in cancer therapy. In ovarian and colorectal cancer, pre-screening for a set of EMT marker genes provides useful diagnostic power for the prediction of chemotherapy response in patients^26^. Based on the classification of intermediate EMT states, a population of ovarian cancers that exhibits intermediate mesenchymal differentiation was most sensitive to the Src kinase inhibitor saracatinib, which induced epithelial features and decreased the stem-like properties of the tumour cells^3^. Src and its associated focal adhesion kinase provide resistance of mesenchymal variants in non-small cell lung cancer to chemotherapeutic drugs, and the Src inhibitor dasatinib renders these tumours susceptible to anti-cancer agents such as erlotinib^42^. Attempts to define novel compounds that could interfere with EMT have reconfirmed major established inducers of EMT, such as TGF-β receptors, the Src kinase and MAPKs^27^. A screen of natural products for their ability to re-epithelialise metastatic and highly mesenchymal breast cancer cells identified the triterpenoid sarasinoside A, which induced intercellular tight junction and activation of the small GTPase Rap1^43^. An independent imaging-based screen for lung adenocarcinoma EMT inhibitors identified methacycline, a compound that interfered with specific MAPK pathways acting downstream of TGF-β; *in vivo*, methacycline blocked the lung fibrosis induced by bleomycin, without perturbing central responses to TGF-β signalling in various stromal cell types^28^.

In conclusion, our high-content imaging screen identified chemical agents that can be used for studies aimed at deciphering the cell biological parameters that specify intermediate, or rather, late stages in the plasticity progression. In addition, the new evidence that certain nuclear receptors protect epithelial cells from pro-fibrotic evolution and also prevent fibroblasts from differentiating towards myofibroblasts, provides an example of how crosstalk between TGF-β signalling and nuclear receptors can generate new avenues for basic and translationally applicable future investigations.

## Methods

### Cell transfection

AG1523 fibroblasts were seeded at 50% confluence in 6-well culture dishes and transfection was performed using Silentfect (Bio-Rad Laboratories AB, Solna, Sweden) as per the manufacturer’s instructions. Control siRNA, LXR-α or LXR-β siRNA were added to the cells at 50 nM for 24 h at which point the medium was changed to serum-free medium containing 5 ng/ml TGF-β or vehicle. Cells were maintained for an additional 24 h in TGF-β and then harvested for RT-PCR or immunoblotting. For luciferase and shRNA silencing assays, AG1523 fibroblasts were transfected as above except that Lipofectamine 3000 (Invitrogen/Life Technologies Corp., Foster City, CA, USA) replaced Silenfect.

MEFs were seeded at 70% confluence in 6-well dishes and transfection was performed using Fugene HD (Roche Diagnostics Scandinavia AB, Bromma, Sweden) according to the manufacturer’s protocol (6:1 (v/v) Fugene to DNA ratio). Twenty four hours after transfection, cells were stimulated with TGF-β for 72 h prior to harvesting lysates for immunoblot of fixing cells for immunofluorescence.

### Immunoblotting

Following the indicated treatments, cells were washed once in ice-cold PBS and proteins were collected in lysis buffer (20 mM Tris-HCl pH 8.0, 1% NP-40, 150 mM NaCl, 2 mM EDTA) supplemented with protease inhibitor cocktail (Roche Diagnostics Scandinavia AB, Bromma, Sweden) and cleared by centrifugation at 14,000x g at 4 °C for 10 min. Lysate protein concentration was measured by a Bradford assay. Equal protein amounts from each sample were separated with sodium dodecyl sulphate-polyacrylamide gel electrophoresis and transferred to a nitrocellulose membrane. Membranes were blocked for 1 h in 5% milk/TBS-T and incubated overnight at 4 °C with primary antibody in TBS-T. Following 3 washes in TBS-T, corresponding HRP-conjugated secondary antibody (Invitrogen/Life Technologies Corp., Foster City, CA, USA) was added in TBS-T at a concentration of 1:20,000 (anti-mouse) or 1:40,000 (anti-rabbit) and incubation prolonged for 1 h at room temperature. Antibody binding was visualised with the enhanced chemiluminescence detection system (Thermo Fischer Scientific Inc., Waltham, MA, USA). Images were captured with a Fuji scanner using the AIDA software (Fuji Inc.) and band intensities were calculated using Photoshop CS3.

### Immunofluorescence and direct fluorescence microscopy

Immunofluorescence experiments were performed on cells seeded onto sterile glass cover slips in 6-well culture dishes. Following the indicated treatments, cells were fixed for 20 min in 3.7% v/v para-formaldehyde in PBS, permeabilised with 0.1% Triton X-100 in PBS for 20 min, blocked for 30 min with 1% BSA in PBS and incubated overnight at 4 °C with the indicated primary antibody at a concentration of 1:500 in 1% w/v BSA. Following primary antibody binding, cells were incubated with anti-mouse Alexa-fluor488 conjugated secondary antibody (Invitrogen/Life Technologies Corp., Foster City, CA, USA) at a concentration of 1:1,000 in 1% w/v BSA for 1 h at room temperature in the dark. To visualise F-actin, permeabilised cells were stained for 20 min with phalloidin conjugated to Alexa-fluor594 (Invitrogen/Life Technologies Corp., Foster City, CA, USA). A minimum of three washes was performed between each of the above mentioned steps. Following the last set of washes, cover slips were placed onto glass slides with VectaShield HardSet mounting medium containing DAPI (Vector Laboratories, Life Technologies Corp., Foster City, CA, USA) for visualisation.

### Real Time RT-PCR

Total RNA was analysed by quantitative RT-PCR as described^14^, using specific PCR primers (see Extended view). A two-tailed paired Student’s t-test (significance at P value < 0.05) was used to compare TGF-β-inducible levels after control or LXR-specific knockdown in triplicate determinations.

## Additional Information

**How to cite this article**: Carthy, J. M. *et al*. Chemical regulators of epithelial plasticity reveal a nuclear receptor pathway controlling myofibroblast differentiation. *Sci. Rep.* **6**, 29868; doi: 10.1038/srep29868 (2016).

## Supplementary Material

Supplementary Information
