# Supplementary material for: Chemical regulators of epithelial plasticity reveal a nuclear receptor pathway controlling myofibroblast differentiation
Source: Sci Rep. 2016 Jul 19;6:29868. doi: 10.1038/srep29868 (PMC4949434; doi:10.1038/srep29868)
Supplement: Supplementary Information [file srep29868-s1.pdf]

Supplementary Information

Screening for chemical regulators of epithelial plasticity points to a nuclear receptor pathway controlling myofibroblast differentiation

Jonathon Carthy, Martin Stöter, Claudia Bellomo, Michael Vanlandewijck, Angelos Heldin, Anita Morén, Dimitris Kardassis, Timothy C. Gahman, Andrew K. Shiau, Marc Bickle, Marino Zerial, Carl-Henrik Heldin and Aristidis Moustakas

Supplementary Methods

Cell culture and treatment. Human HaCaT keratinocytes, human prostate cancer PC3U cells, human lung epithelial HPL1 cells and culture conditions, were as described[1,2]. Immortalized human mammary epithelial HMLE, MCF10A parental cells and their derivative Ras-transformed MCF10AneoT (MII) cells, human luminal and myoepithelial mammary cells, were maintained in DMEM/F12 supplemented with 5% fetal bovine serum (FBS), 20 ng/ml epidermal growth factor, 100 ng/ml cholera toxin, 0.5 µg/ml hydrocortisone, 10 µg/ml insulin, 100 U/ml penicillin, and 100 µg/ml streptomycin. Prof. Isabel Fabregat, Idibell Barcelona, kindly provided Hep3B cells. Hep3B were cultured and maintained in Minimum Essential Medium Eagle (MEM, Gibco, Life Technologies Corp., Foster City, CA, USA), supplemented with 10% FBS (Biowest SAS, Nuaillé, France) and penicillin-streptomycin (Sigma-Aldrich Sweden AB, Stockholm, Sweden), glutamine (Sigma-Aldrich Sweden AB, Stockholm, Sweden) and non-essential amino acids (Sigma-Aldrich Sweden AB, Stockholm, Sweden). Prof. Wolfgang Mikulits, Medical University of Vienna, Austria, kindly provided SNU423 cells. SNU423 were cultured and maintained in Roswell Park Memorial Institute 1400 medium (RPMI-1400 Gibco, Life Technologies Corp., Foster City, CA, USA), supplemented with 10% FBS (Biowest SAS, Nuaillé, France) and penicillin-streptomycin (Sigma-Aldrich Sweden AB, Stockholm, Sweden) and glutamax (Gibco, Life Technologies

Corp., Foster City, CA, USA). Immortalized human HTERT fibroblasts, AG1523 foreskin fibroblasts and MEFs were provided by Paraskevi Heldin and Johan Lennartsson at our Institute, respectively, and were cultured in 10% FBS/DMEM with penicillin-streptomycin. Human lung adenocarcinoma A549 cells were cultured in 10% FBS/DMEM with penicillin-streptomycin. Cells were maintained in a humidified incubator at 37 °C and 5% CO_2.

Cell treatments with TGFβ and compounds were done in 10% FBS unless otherwise specified. Cells were treated with 5 ng/ml TGF-β (or vehicle control) and compound (or DMSO) was added at a concentration between 2.5-10 μM and maintained for 24-96 h. Exact experimental conditions are indicated in the text. In certain experiments the TGF-β receptor kinase inhibitor GW6604 was used at 3.3 μM as a positive control.

Reagents. Human *LXRα*-specific siRNA, ON-TARGETplus SMARTpool reagent NR1H3 L-003413-00 (Acc. No. Q13133, Q9Z0Y9, Q62685), human LXRβ-specific siRNA, ON-TARGETplus SMARTpool reagent NR1H2 L-003412-02 (Acc. No. P55055, Q60644, Q62755, P34021), and control siRNA against the *luciferase* reporter vector pGL2 (Acc. No. X65324) were from Dharmacon Research, Inc., Boulder, CO.

Expression vectors pcDNA3, pSG5-hLXRα, pSG5-hLXRβ (both kind gifts of K. Steffensen, Karoniska Institute, Stockholm) were amplified and purified from *E. coli*. The pCAGA$_{12}$-luc reporter and the pCMV-β-gal reporter used for normalisation of transfection efficiency have been described before[1,2] . The pSuper empty vector (a gift from R. Agami, Netherland Cancer Institute, Amsterdam) and the pSuper-LXRα and pSuper-LXRβ shRNA vectors were previously described[3].

Recombinant mature human TGF-β1 was from PeproTech EC Ltd. (London, UK) and Biosource Inc. (Camarillo, CA, USA). The TGF-β1 isoform used throughout this study is

referred to as TGF-β; it was dissolved in 4 mM HCl/0.1% fatty acid-free bovine serum albumin (BSA).

Chemical compounds. Compounds were obtained from the following sources: 24(S)-hydroxycholesterol (EPM-1), 24(S),25-epoxycholesterol and SR12813 were purchased from Enzo Life Sciences, Inc., Farmingdale, NY, USA; estradiol valerate (EPM-2), bumetanide (EPM-3), lanosterol (EPM-4), 4-nonylphenol (EPM-5), 22(R)-hydroxycholesterol, T0901317, estradiol, DES, tamoxifen, progesterone, mifepristone (RU486), PCN, TCOBOP, CITCO and azaserine were purchased from Sigma-Aldrich Co. St. Louis, MO, USA; acivicin (EPM-6) was purchased from LKT Laboratories, St. Paul, MN, USA; EPM-10 was purchased from Enamine LLC, Monmouth Jct., NJ, USA; EPM-11, EPM-12, EPM-13, EPM-14 and EPM-15 were purchased from ChemDiv, San Diego, CA, USA; EMP-16 was purchased from Key Organics Ltd, Camelford, UK; GSK3987 was purchased from EMD Millipore, Billerica, MA, USA; WYE672 was purchased from Axon Medchem BV, Groningen, The Netherlands; 24(R)-27-hydroxycholesterol was purchased from Cayman Chemical Co., Ann Arbor, MI, USA; GW3965 was purchased from Selleckchem, Houston, TX, USA; Tularik antagonist (cmpd 54) and GSK antagonist (GSK 2033) were custom synthesized by Sundia Meditech, Shanghai, P.R. China; DON was purchased from Bachem Inc., Torrance, CA, USA; decoyinine was purchased from Santa Cruz Biotechnology, Santa Cruz, CA, USA.

High-content screen of chemical compounds. HaCaT cells were seeded in 3% FBS/DMEM and 5 ng/ml TGF-β into 384 well plates (3,000 cells in 45 μl/well) using the drop dispenser 'MultiDrop' or 'WellMate' (ThermoScientific, Thermo Fischer Scientific Inc., Waltham, MA, USA). In column 2, cells were seeded without TGF-β (see Fig. S2D for experimental layout of a representative 384-well plate). After 24 h compounds and controls were administered to

the cells by adding 5 µl of a 1:100 dilution of the DMSO solution in medium using the 'Freedom Evo' pipetting robot (Tecan Group Ltd, Männedorf, Switzerland) equipped with a 96-head piece and with disposable tips (final concentration was depending on the library, but in most cases 10 µM or 16.6 µM). As positive controls GW6604 or LY-364947 were added in five concentrations (0.1, 0.3, 1.0, 3.0 and 10.0 µM) and staurosporine was added in two concentrations (0.001 and 0.1 µM). As negative control DMSO was added (0.1% v/v, n=29). The cells were washed 72 h after seeding (48 h after compound addition) with PBS using the plate washer 'PW384' (Tecan Group Ltd, Männedorf, Switzerland) and fixed for 30 min by addition of para-formaldehyde (final concentration 4% (w/v) in PBS).

The proof-of-concept screen in 96-well format was done by seeding cells (3,000 in 100 µl/well) without TGF-β, replacing the medium 24 h later with medium containing TGF-β and adding 48 h after seeding seven concentrations of compounds in 50 µl of a 1:3 dilution of the DMSO solution (final concentration 41 nM – 30 µM) using a manual multi-channel pipette. As positive control GW6604 (3.3 µM) was added and as negative control DMSO was added (0.3% v/v, n=6). Ninety six hours after seeding (48 h after compound addition) the cells were washed once with PBS using a manual 8-needle adaptor and an automated multi-channel pipette. After fixation for 30 min with para-formaldehyde (final concentration 4% (w/v) in PBS), cells were washed twice with PBS. All further steps were performed manually in the same way as described further down for the robotic liquid handling, except that multi-channel pipettes and the 8-needle adaptor were used instead of the robotic automation.

For the robotic immunostaining procedure in 384-well plates the above mentioned automation (dispenser, pipetting robot and plate washer) were used. For immunostaining of fibronectin cells were permeabilised for 10 min with 1% Triton X-100 in PBS, blocked for 1 h with blocking solution (5% FBS/0.1 mM glycine in PBS), and incubated with the primary antibody (anti-fibronectin rabbit polyclonal, cat# F3648, Sigma-Aldrich Sweden AB,

Stockholm, Sweden) using a dilution of 1:1,000 in blocking solution. After washing with PBS, the cells were incubated with a secondary antibody (anti-rabbit Alexa-568, cat# A10042, Molecular Probes, Life Technologies Corp., Foster City, CA, USA) using a dilution of 1:500 in blocking solution. Finally, the cells were washed again with PBS, and nuclei were stained with 1 μg/ml 4',6'–diamidino-2-phenylindole (DAPI) and sodium azide was added (0.02% w/v in PBS) to prevent contamination. Imaging plates were acquired automatically on the HCS microscope 'ArrayScan' (Cellomics/ThermoScientific, Thermo Fischer Scientific Inc., Waltham, MA, USA) using a 10× objective (NA = 0.3) taking 9 fields with 2 channels; for DAPI the filter was set to XF93-Hoechst and for fibronectin-568 the filter was set to XF93-TRITC.

For the counter-screen, cells were seeded without TGF-β, and 24 h later, they were treated with compounds for 2 h, before TGF-β was added to the cells. After 1 h of TGF-β treatment cells were fixed and stained as described above using the anti-p-Smad2 (rabbit polyclonal antibody, home-made, LICR-Uppsala, Sweden)[1,2] as a primary antibody. Plates were imaged as described above.

Image and data analysis of the high-content screen. Images of HaCaT cells taken on the 'ArrayScan' HCS platform were automatically analysed using the module 'Morphology Explorer' of the 'BioApplications' software package (Cellomics/ThermoScientific, Thermo Fischer Scientific Inc., Waltham, MA, USA). Two algorithms were optimised; the first was segmenting single nuclei, the second segmenting groups of cells as colonies. In total, nearly 500 measurement parameters were calculated. With the help of the open-source data analytics software KNIME[4] and the plug-ins HCS-Tools and Scripting Integration, that were necessary to handle screening data[5], all parameters were evaluated during assay development for robustness, repeatability, and redundancy (manuscript in preparation). Finally, 18 parameters

were selected to generate a multi-parametric profile for each compound (Fig. S3). Next the measurements of each plate were normalised by calculating the robust percentage-of-control (.poc)[6]:

x_{rcpm}.poc = x_{rcpm}/median(x_m[DMSO]$_p$)*100, where x_{rcpm} is the measurement x of parameter m of a well in row r, column c, and plate p, and x_m[DMSO]$_p$ are the measurements x of parameter m of all DMSO negative control wells per plate p (here n = 29). Then all parameters were normalised for their distribution by calculating the z-score (.zscore):

x_{rcpm}.zscore = (x_{rcpm} – mean(x_m[DMSO]))/sd(x_m[DMSO]), where x_{rcpm} is the plate normalised measurement x (.poc) of parameter m of a well in row r and column c and plate p, x_m[DMSO] are the plate normalised measurements x (.poc) of parameter m of all DMSO negative control wells of the screen (here n = 5133), and sd is the standard deviation.

Last, a profile normalisation was done for clustering the data (.lennorm):

x_{rcp}.eucldist = $\sqrt{}$($x_{rcpm1}^2 + x_{rcpm2}^2 + \ldots + x_{rcpmn}^2$), where x_{rcp}.eucldist is the Euclidian distance x of the profile of a well in row r, column c, and plate p, and x_{rcpm} is the plate normalised, zscore measurement x (.poc.zscore) of parameter m of a well in row r, column c, and plate p, with m_1 as the first parameter, and m_n as the last parameter of the profile (here n = 18).

x_{rcpm}.lennorm = x_{rcpm}/x_{rcp}.eucldist, where each parameter m is normalised to the Euclidian distance of the profile.

The profile normalised data were used to cluster all data with the k-means algorithm and evaluation of all control wells indicated that k = 5 performed best for this data set. The cluster 1, containing most of the EMT positive control wells, was filtered for weak phenotypes using a threshold of 15 on the Euclidian distance. Control wells and wells missing an annotation were removed from the list of hits and after aggregation of repeated plates 93 hits were defined.

The counter-screen was analyzed with the module 'CytNuc Translocation' of the 'BioApplications' and only one measurement parameter was selected for the p-Smad2 channel (CytNuc_MEAN_CircRingAvgIntenRatioCh2). The analysis of the verification and the validation screen was done as described above, except that the p-Smad2 parameter was considered separately. Taking these data together, a final list of 16 compounds, as shown in Fig. S2D, was obtained.

Antibodies and dilutions used for immunoblotting. Antibodies used for immunoblotting were as follows: fibronectin 1:10,000 (Sigma-Aldrich, F3648), E-cadherin 1:5,000 (BD Biosciences, 610182), N-cadherin 1:50,000 (BD Biosciences, 610920), claudin-3 1:500 (Zymed, 34-1700), p-Smad2 1:1,000 (rabbit polyclonal anti-phospho-Smad2 produced in house)[1,2], p-Smad3 1:1,000 (Cell Signaling, phospho-Smad3 (Ser423/425) (C25A9) product number 9520S), smooth muscle α-actin 1:5,000 (Santa Cruz Biotechnology, product number sc-32251), LXRα 1:1,000 (Abcam, ab41902), LXRβ 1:1,000 (ThermoScientific Pierce, PA1-333), Gapdh 1:50,000 (Ambion, AM4300), CAR 1:2,000 (homemade, epitope used SIV, kind gift of Teresa Vincent, Karolinska Institute, Stockholm), vimentin 1:1,000 (13.2, Sigma-Aldrich), PAI-1 1:5,000 (plasminogen activator inhibitor 1, BD Pharmingen/Transduction Laboratories, BD Biosciences, Stockholm, Sweden), pan-cytokeratin 1:500 (Sigma-Aldrich), α-tubulin 1:5,000 (Santa Cruz Inc. USA).

Fluorescence microscopy. A Zeiss Axioplan 2 immunofluorescence microscope was used with the Zeiss 20×, 40× or 63× objective lens and photographing at ambient temperature in the presence of immersion oil. Images were acquired with a Hamamatsu C4742-95 CCD digital camera and the acquisition software QED Camera Plugin v1.1.6 (QED Imaging Inc.)

and Volocity® (PerkinElmer Waltham, MA, USA). Images were processed with Adobe Photoshop CS3 to reduce file size.

Three-dimensional hepatosphere culture and protein extraction. SNU423 cells were seeded at a density of 1.2×10^3 per well in a 96-well plate of the GravityPLUS™ Hanging Drop System (InSPHERO) in RPMI-1400/10% FBS, for 72 h and in the absence or presence of specific EPM compounds at a final concentration of 10 μM. Hepatospheres were photographed directly in the InSPHERO plates using a Zeiss Axioplan 2 microscope and its 10× objective lens and photographing at ambient temperature in the absence of immersion oil. Images were acquired with a Hamamatsu C4742-95 CCD digital camera and the acquisition software QED Camera Plugin v1.1.6 (QED Imaging Inc.) and Volocity® (PerkinElmer Waltham, MA, USA). Images were processed with Adobe Photoshop CS3 to reduce file size. Hepatospheres were retrieved and their proteins were extracted using a lysis buffer made of 20 mM Tris-HCl, pH 7.4, 150 mM NaCl, 150 mM EDTA, 0.5% v/v Triton X-100, 0.5% v/v Na-deoxycholate, 0.25 mM LiCl, 5 mM NaF, 1 mM Na_3VO_4, and Complete™ protease inhibitor chocktail (Roche Diagnostics Scandinavia AB, Bromma, Sweden). In order to facilitate protein retrieval, hepatospheres dissolved in the lysis buffer were snap-freezed in liquid nitrogen and slowly thawed in ice for three repeated cycles, prior to centrifugation at 20,000 rpm at 4 °C and collection of the soluble supernatant that was analysed by gel electrophoresis and immunoblotting.

Collagen Gel Contraction Assay. Twelve-well culture dishes were coated with 1% BSA and incubated overnight at 37°C to create a surface that prevents gels from attaching to the dishes. AG1523 cells were trypsinised, counted and seeded into a 1 mg/ml Type I collagen solution (PureCol, Advanced BioMatrix) in serum-free medium at a concentration of 1×10^5 cells/ml.

The collagen/cell suspension was vortexed, and 1 ml per well was added to the BSA-coated dishes and the solution was allowed to polymerise for 45 min at 37°C. Fresh medium containing the indicated treatment conditions was then added to the solidified collagen gels and plates were returned to the incubator. Collagen gel contraction was monitored over a period of 72 h and the surface area of contracted gels was measured using ImageJ software. In certain experiments, the protocol was modified by first transfecting MEFs with empty or LXRα expression vector for 24 h prior to trypsinising and counting the cells for the assay. In these experiments cells were maintained in full serum throughout and treated accordingly.

Real Time RT-PCR primer sequences. The gene-specific PCR primers used were:
for *LXR-α,* forward 5'-CCACCGAGACTTCTGGACAGG-3' and reverse 5'-GCAGAGTCAGGAGGAATGTCAG G-3';
for *LXR-β,* forward 5'-AGCACAGACTGGGTCATCCC-3' and reverse 5'-AGCACGTTG TAGTGGAAGCC-3';
for *SNAI1*, forward 5′-CACTATGCCGCGCTCTTTC-3′ and reverse 5′-GCTGGAAGGTAAACTCTGGATTAGA-3′; for *SNAI2*, forward 5′-AGACCCTGGTTGCTTCAAGGA-3′and reverse 5′-CTCAGATTTGACCTGTCTGCAAA-3′; for *TWIST1*, forward 5′-GTCCGCGTCCCACTAGCA-3′and reverse 5′-TTCTCTGGAAACAATGACATCTAGGT-3′; for *HMGA2*, forward 5′-GACGTCGGGCATTCATATAGG-3′and reverse 5′-TTGGTGTTCTAAACAGAGGATTCACT-3′; for *glyceraldehyde-3'-phosphate dehydrogenase* (*GAPDH*), forward 5'-TGTGTCCGTCGTGGATCTGA-3' and reverse 5'-CCTGCTTCACCACCTTCTTGA-3'; *GAPDH* was used as the reference gene.

Luciferase assay. AG1523 fibroblasts were transiently transfected with the TGF-β/Smad-responsive construct $CAGA_{12}$-luc for 24 h prior to stimulation with TGF-β for 18 h. pCMV-β-gal was co-transfected as control for normalisation. The cells were lysed in lysis buffer containing 5 mM Tris-phosphate (Tris-HCl/KH_2PO_4) buffer pH 7.8, 2 mM dithiothreitol, 2 mM CDTA (trans-1,2-diaminocyclohexane-N,N,N',N' tetra-acetic acid), 5% glycerol and 1% Triton X-100. The β-Galactosidase assay was performed by mixing the cell lysate with 100 mM sodium phosphate pH 7.3, 1 mM $MgCl_2$, 50 mM β-mercaptoethanol and 0.67 mg/ml of ONPG (o-Nitrophenyl β-D-Galactopyranoside) and the absorbance was monitored at 420 nm. Luciferase reporter assays were performed with the enhanced firefly luciferase assay kit from either BD PharMingen, Inc. or from Biotium Inc., according to the protocol of the manufacturers. Normalised promoter activity data are plotted in bar graphs that represent average values from triplicate determinations with standard deviations. Each independent experiment was repeated at least three times.

Supplementary References

1. Kowanetz, M., Valcourt, U., Bergström, R., Heldin, C.-H. & Moustakas, A. Id2 and Id3 define the potency of cell proliferation and differentiation responses to transforming growth factor β and bone morphogenetic protein. *Mol. Cell. Biol.* **24**, 4241-4254 (2004).
2. Valcourt, U., Kowanetz, M., Niimi, H., Heldin, C.-H. & Moustakas, A. TGF-β and the Smad signaling pathway support transcriptomic reprogramming during epithelial-mesenchymal cell transition. *Mol. Biol. Cell* **16**, 1987-2002 (2005).
3. Thymiakou, E., Zannis, V. I. & Kardassis, D. Physical and functional interactions between liver X receptor/retinoid X receptor and Sp1 modulate the transcriptional induction of the human ATP binding cassette transporter A1 gene by oxysterols and retinoids. *Biochemistry* **46**, 11473-11483 (2007).
4. Berthold, M. R. *et al.* KNIME: The Konstanz Information Miner. *Data Analysis, Machine Learning and Applications*, 319-326 (2008).
5. Stöter, M. *et al.* CellProfiler and KNIME: open source tools for high content screening. *Methods Mol. Biol.* **986**, 105-122 (2013).
6. Malo, N., Hanley, J. A., Cerquozzi, S., Pelletier, J. & Nadon, R. Statistical practice in high-throughput screening data analysis. *Nat. Biotechnol.* **24**, 167-175 (2006).

Supplementary tables

Compound Group	EPM number	Common name	HCS pheno type	HaCaT EMT	HaCaT MET	A549 EMT	A549 MET	MCF10A MII EMT	MCF10A MII MET	Hep3B EMT	Hep3B MET	SNU423 spheres	SNU423 MET	Fibroblast FN	Fibroblast αSMA
Simple heterocyclic/	3	Bumetanide	+	-	-	-	-	+	-	+	-	n.d.	n.d.	+	-
carboxylic acid	6	Acivicin	+	+	-	-	-	+	-	+	-	n.d.	n.d.	+	+
analogs	8		+	n.d.	n.d.	n.d.	n.d.	n.d.	n.d.	n.d.	n.d.	n.d.	n.d.	n.d.	n.d.
	9		+	n.d.	n.d.	n.d.	n.d.	n.d.	n.d.	n.d.	n.d.	n.d.	n.d.	n.d.	n.d.
	12		+	+	-	-	-	+	-	+	-	n.d.	n.d.	+	+
Steroidal/alkyl	1	24(S)-Hydroxycholesterol	+	+	-	+	-	-	+	+	-/+	+	+	+	+
analogs	2	Estradiol valerate	+	+	-	+	-	-	-	+	-	n.d.	n.d.	+	+
	4	Lanosterol	+	+	-	+	-	+	-	+	-	n.d.	n.d.	+	+
	5	4-Nonylphenol	+	+	-	+	-	+	-	+	-	n.d.	n.d.	+	+
	7	Totaralolal	+	+	-	+	-	+	-	+	-	n.d.	n.d.	+	+
Extended multi-ring	10		+	-	+	-	+	-	+	+	-	+	+	+	-
analogs	11		+	+	-	-	-	+	+	+	-	n.d.	n.d.	+	+
	13		+	+	+	+	+	+	+	+	-	+	-	+	+
	14		+	+	-	+	-	+	+	+	-	n.d.	n.d.	+	-
	15		+	+	-	+	-	+	+	+	-	n.d.	n.d.	+	+
	16		+	n.d.	n.d.	n.d.	n.d.	n.d.	-	n.d.	n.d.	n.d.	n.d.	n.d.	n.d.

Table S1. Summary of the activity results of 13 compounds on various cell-based assays. The table lists the three classes of compounds; their EPM numbers; those with known alternative names; the cumulative results from: a) the high content screen (HCS), b) the EMT profile (several mesenchymal markers) in HaCaT cells, c) the epithelial marker and adhesion profile (mesenchymal-epithelial transition, MET) in HaCaT cells, d) the EMT profile (several mesenchymal markers) in A549 cells, e) the epithelial marker and adhesion profile (mesenchymal-epithelial transition, MET) in A549 cells, f) the EMT profile (several mesenchymal markers) in MCF10A MII cells, g) the epithelial marker and adhesion profile (mesenchymal-epithelial transition, MET) in MCF10A MII cells, h) the EMT profile (several mesenchymal markers) in Hep3B cells, i) the epithelial marker and adhesion profile (mesenchymal-epithelial transition, MET) in Hep3B cells, j) hepatosphere effects in SNU423 cells, k) the mesenchymal marker profile (MET) in SNU423 cells, l) the fibronectin (FN) profile in human fibroblasts, m) the α-SMA profile in human fibroblasts. The summary table tabulates results as clear positive effect (+) or clear negative effect (-). Within those with a positive effect (+), different compounds have shown different potency depending on the cell model studied. Compounds that were not studied are listed as n.d. (not determined).

Supplementary Figure Legends and Results

Supplementary Figure 1 Carthy et al

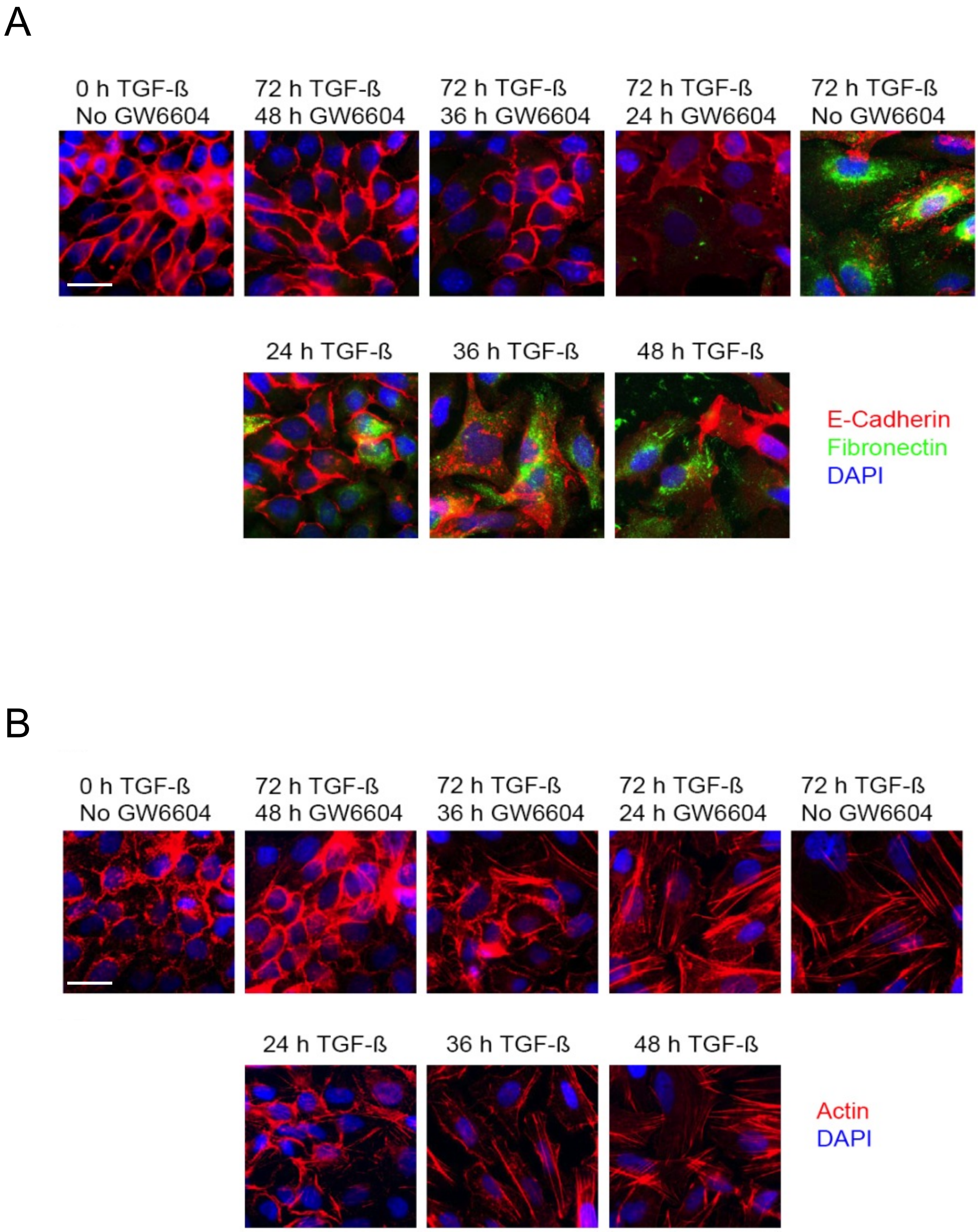

Figure S1. EMT of HaCaT cells caused by TGF-β and reversion by GW6604. (**A**) HaCaT cells were stimulated with 5 ng/ml TGF-β for the indicated times, in the absence (DMSO vehicle) or presence of 3.3 μM GW6004. Indirect immunofluorescence was performed using anti-E-cadherin and anti-fibronectin antibodies, while DNA was stained using DAPI. Note the gradual loss of E-cadherin-positive adherens junctions and the gradual gain of cytoplasmic/vesicular and extracellular fibronectin. At the bottom row, photomicrographs from the same experiment in the absence of GW6604 show the kinetics of EMT establishment. A white bar indicates 10 μm. (**B**) Actin cytoskeleton reorganization in HaCaT cells responding to TGF-β or GW6604 as in panel A. Cells were labeled with TRITC-phalloidin and DAPI. Note the clear transition between a cortical actin arrangement and a stress fiber pattern. A white bar indicates 10 μm.

Supplementary Figure 2 Carthy et al

A

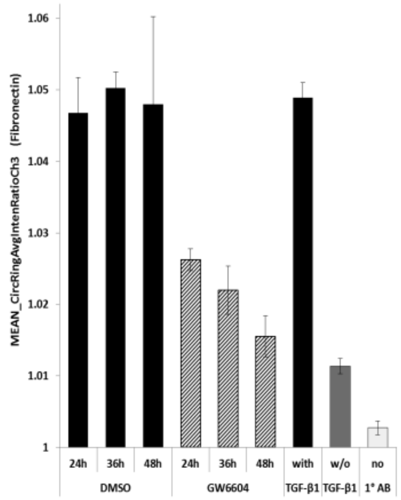

B

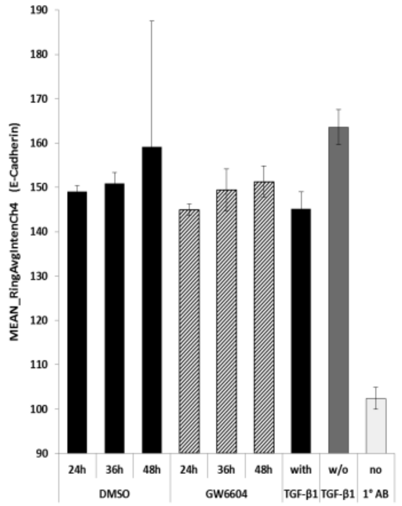

C

S compound_name	SDF supplier_molecule	I total_hit_count	S concentration [µM] (count)
TGF-b RI Kinase Inhibitor (LY-364947)	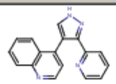	12	10.0(2), 3.3(2), 1.1(2), 0.37(2), 0.1234(3), 0.0412(1)
GW6604	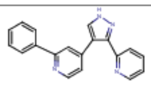	7	10.0(2), 3.3(2), 1.1(2), 0.37(1)
GSK-3b Inhibitor IX	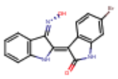	4	1.1(2), 0.37(2)
Oxindole I	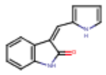	2	10.0(2)

D

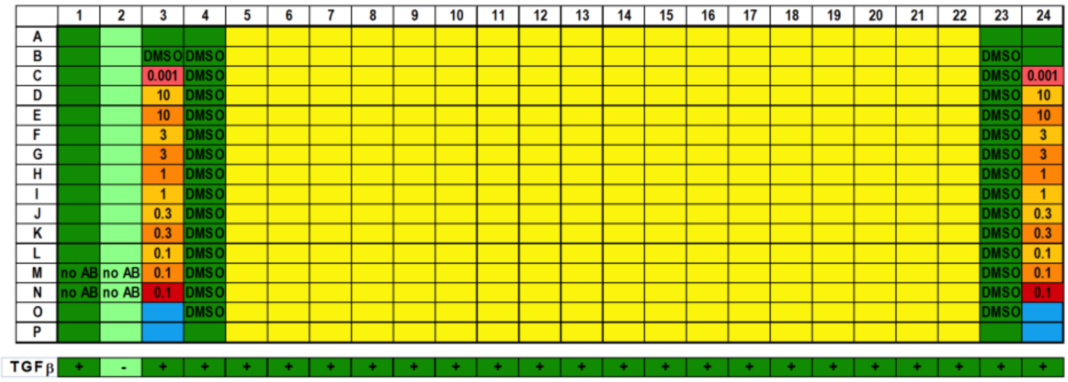

	GW6604	LY-364946
10µM	+++	+++
3µM	+++	+++
1µM	+	+++
0.3µM	+/-	++
0.1µM	-	++

Strength of phenotype

	library		
	no TGFβ		EMT positive control (reference wells)
	TGFβ		EMT negative control
	GW6604	10 - 0.1µM	sentitivity control
	LY-364947	10 - 0.1µM	sentitivity control
	no DMSO		DMSO toxicity control
	Staurosporine	0.1µM	20% apoptosis control
	Staurosporine	0.001µM	95% cell death control
DMSO	negative controls used for normalization		

Figure S2. Optimisation of HCS assay in HaCaT cells using GW6604. (**A, B**) HaCaT cells stimulated with 5 ng/ml TGF-β for 72 h were co-treated either with 3.3 μM GW6604 or with DMSO for the indicated time. Fibronectin and E-cadherin were measured by immunostaining and image analysis using the ArrayScan HCS-reader. Measurements with highest Z'-factors are displayed. Controls without primary antibodies give the absolute background level. (A) Treatment with GW6604 for 48 h reduced expression of fibronectin - measured by the ratio of nuclear to cytoplasmic intensity - close to the levels of unstimulated control (w/o TGF-β). (B) E-cadherin expression measured by cytoplasmic intensity was only slightly increased after treatment with GW6604 for 48 h revealing a worse signal-to-noise ratio than fibronectin. (**C**) As a poof-of-principle screen, a set of 78 compounds (mostly protein kinase inhibitors) were screened at 8 concentrations (4.6 nM – 10 μM) using the high-content screening setup described here. In brief, HaCaT cells stimulated with 5 ng/ml TGF-β for 72 h were co-treated with compounds for 48 h, fixed, immunostained for fibronectin and analysed with the HCS platform ArrayScan. The table summarises hits of this pilot screen in which two TGF-β type I receptor kinase inhibitors (LY-364947 and GW6604) were found as hits at several concentrations. After further analysis, including dose-response curves, GSK-3β inhibitor IX and Oxindole I were not followed up as hits. (**D**) Layout for screen in 384-well plate format. Each plate contained DMSO negative control wells (n=29) for plate normalisation, unstimulated wells without TGF-β (no TGFβ/TGFbm, n=12), negative control wells for immunostaining (no AB/Abm, n=2x2) and negative untreated wells (no DMSO, n=4). As positive controls, TGF-β type I receptor kinase inhibitors GW6604 and LY-364947 were added at 5 concentrations (0.1, 0.3, 1, 3 and 10 μM, each n=2) and staurosporine was added at 2 concentrations (0.001 and 0.1 μM, each n=2). The strength of phenotype (i.e. suppression of fibronectin intensity and enhanced colony density) is also tabulated for each TGF-β type I

receptor kinase inhibitor concentration below the layout. Some outer wells of negative controls were excluded from the analysis.

Supplementary Figure 3 Carthy et al

A

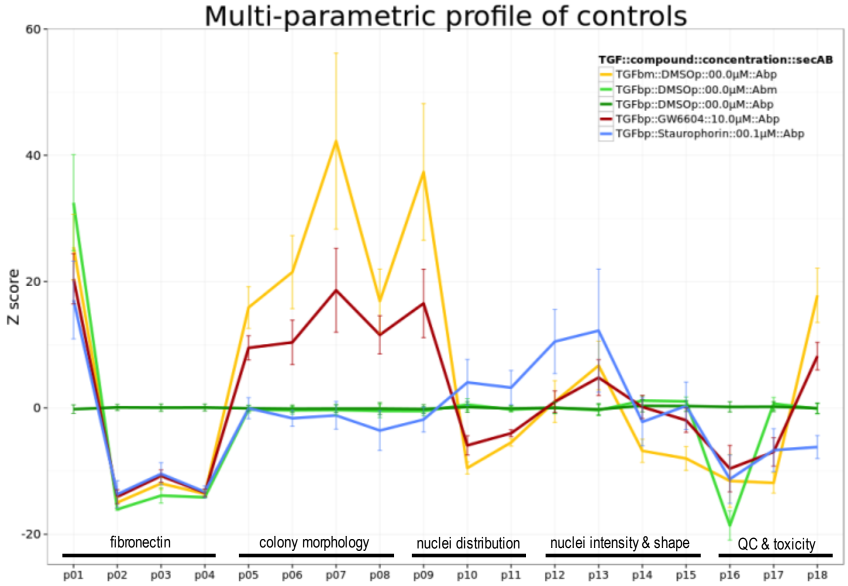

B

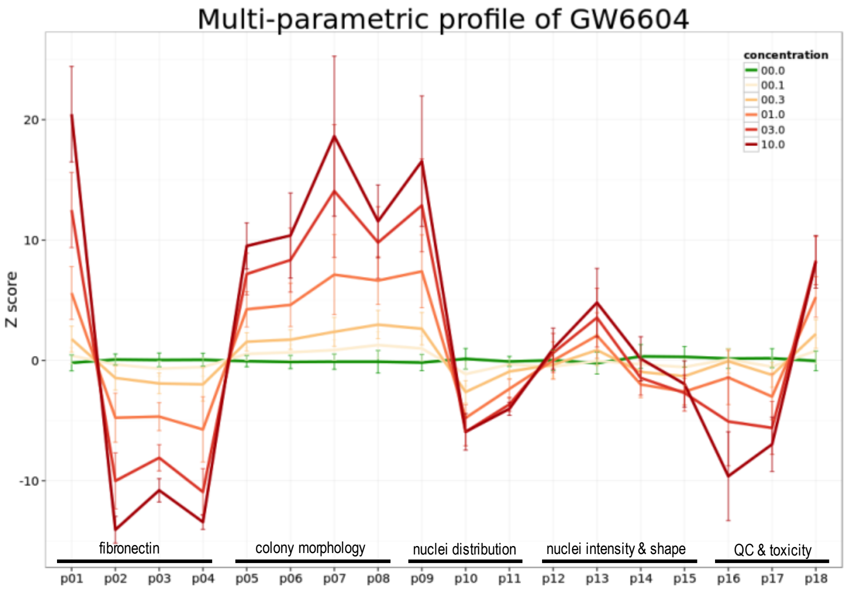

C

parameter number	parameter name	group
p01	Colony_MEAN_ContrastCoocIntenCh3.poc.zscore	fibronectin
p02	Colony_%LOW_ContrastCoocIntenCh3.poc.zscore	
p03	Colony_MEAN_EntropyIntenCh3.poc.zscore	
p04	Colony_%HIGH_EntropyIntenCh3.poc.zscore	
p05	Colony_MEAN_ObjectLengthCh1.poc.zscore	colony morphology
p06	Colony_SD_ObjectLengthCh1.poc.zscore	
p07	Colony_MEAN_ObjectPerimCh1.poc.zscore	
p08	Colony_SD_ObjectConvexHullPerimRatioCh1.poc.zscore	
p09	Colony_MEAN_MemberOutCountCh2.poc.zscore	nuclei distribution
p10	SiCell_MEAN_NeighborMinDistCh1.poc.zscore	
p11	SiCell_SD_NeighborMinDistCh1.poc.zscore	
p12	Colony_MEAN_ObjectSkewIntenCh1.poc.zscore	
p13	Colony_MEAN_ObjectKurtIntenCh1.poc.zscore	nuclei intensity & shape
p14	SiCell_MEAN_ObjectAreaCh1.poc.zscore	
p15	SiCell_MEAN_ObjectLengthCh1.poc.zscore	
p16	Colony_SD_EntropyIntenCh3.poc.zscore	QC & toxicity
p17	Colony_SelectedObjectCount.poc.zscore	
p18	SiCell_SelectedObjectCountPerValidField.poc.zscore	

Figure S3. Representative multi-parametric profiles of the high-content screen for inhibitors of TGF-β-induced EMT. (**A, B**) Multi-parametric profiles of 18 measurement parameters selected to describe phenotype of cells undergoing EMT. These parameters describe the fibronectin expression pattern (4×, specific for EMT and toxicity), the size and morphology of groups of cells forming a colony (4×, specific for EMT vs toxicity), the distribution of nuclei (3×, specific for EMT vs toxicity), the intensity and shape of nuclei (4×, specific for toxicity) and performance of the assay (QC) and toxicity (3×, specific for immunostaining and toxicity). Profiles in (A) display the median of all wells of the entire screen (positive controls: n=354; negative DMSO control: n=8848). The profiles of unstimulated cells (TGFbm, yellow) and GW6604 treated cells (brown) are typical for inhibition or reversion of EMT, the profile of staurosporine (blue) is typical for compound-induced toxicity, the profile of unstained cells (Abm, light green) is typical for inhibition of fibronectin expression without inhibition of EMT and the base line profile (dark green) are the TGF-β (TGFbp) and DMSO treated negative control wells. (**B**) Multi-parametric profiles of five different concentrations (0.1 - 10 μM, light brown to dark brown) of positive control GW6604 (n=354). The profiles indicate that weak to strong phenotypes can be measured using this assay. (**C**) List of 18 parameters selected for analysis of the EMT phenotypic screen. Parameters could be divided into five descriptive groups, as explained in panels A and B.

Supplementary Figure 4 Carthy et al

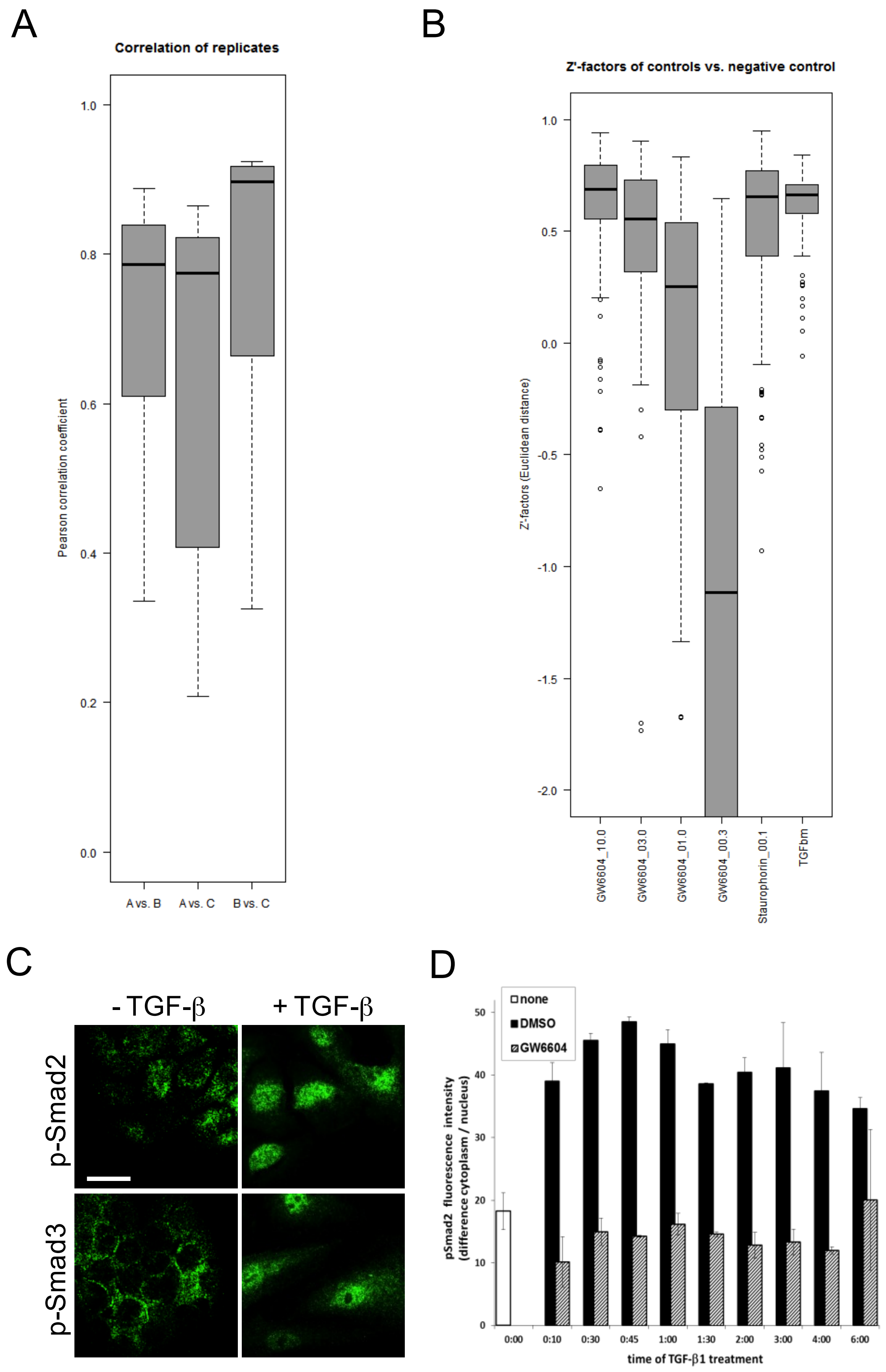

Figure S4. Statistical analysis of the high-content screen and secondary screen against TGF-β inhibitors. (**A**) Box plot of Pearson correlation coefficients of all 18 parameters for three independent repeats. A median correlation of all parameters runs around 0.8 and indicates a very good reproducibility. (**B**) The box plot of Z'-factors of several positive controls vs. the DMSO negative control for all screening plates (n=177) shows that the assay is very robust and reproducible for the strong EMT inhibitor controls (GW6604 (median): 10 µM = 0.69, 3 µM = 0.55) and for the staurosporine control (median: 0.1 µM = 0.66). (**C**) Immunofluorescence microscopy of HaCaT cells for the indicated proteins in cells stimulated with vehicle or 5 ng/ml TGF-β. Phosphorylated Smad2 and Smad3 translocate to the nucleus after TGF-β stimulation (+ TGF-β). Technical conditions were as in Figure S1. (**D**) HaCaT cells stimulated with 5 ng/ml TGF-β for the indicated time were co-treated either with 3.3 µM GW6604 or with DMSO. Treatment with GW6604 inhibited translocation of p-Smad2 into the nucleus. Translocation of p-Smad2 was measured by immunostaining and image analysis (relative intensity) using the ArrayScan HCS-reader, and plotted as the differential in fluorescence intensity between the two compartments. Translocation of p-Smad2 and effect of GW6604 induced inhibition of translocation were highest around 1 h after stimulation.

Supplementary Figure 5 Carthy et al

library code::library plate number::plateRow ::plateColumn	A::prim Screen	B::prim Screen	C::prim Screen	A::primScreen(vector-norm)	B::primScreen(vector-norm)	C::primScreen(vector-norm)	A::verif Screen (cluster VerifScreen)	B::verif Screen (cluster VerifScreen)	A::verif Screen(vector-norm)(clusterVerifScreen)	B::verif Screen(vector-norm)(clusterVerifScreen)	hit verification	library code::library plate number::plateRow ::plateColumn::compound name (Unique concatenate) (Unique concatenate)	CytNuc_MEAN_CircRingAvgIntenRatioCh2.poc.zscore (Mean)	CytNuc_MEAN_CircRingAvgIntenRatioCh2.poc.zscore (Concatenate)
KBI::2::7::20	1	0		24.87	40.43		0	0	7.08	6.67	not confirmed, twice in EMT cluster	KBI::2::7::20_PP2	-0.49	-0.48564670412155003, -0.4985274008392051
CPW::1::6::10	1	1	1	17.96	13.30	9.32	0	0	6.80	5.22	not confirmed, twice in EMT cluster	CPW::1::6::10_	-0.01	0.2359266741731543, -0.247423807885643
CBN::13::2::7	1			16.04			0	0	4.54	2.85	not confirmed, twice in EMT cluster	CBN::13::2::7_	0.04	0.29650866055588804, -0.2074420530921415
CBN::58::14::19	1			17.81			0	0	2.17	3.23	not confirmed, twice in EMT cluster	CBN::58::14::19_	0.05	-0.08091691127404453, 0.1845814577836836
KBI::2::3::16	1	0		20.42	41.68		0	0	6.22	3.98	not confirmed, twice in EMT cluster	KBI::2::3::16_PP1	0.06	0.17749091141059556, -0.0625386771978938
MSD::2::7::7	0	1	1	48.45	29.29	32.97	2	2	39.13	43.78	not confirmed, different phenotype	MSD::2::7::7_LANATOSIDE C	-2.96	-4.394284693264937, -1.521363972255098
MSD::6::13::13	3	3	1	17.43	22.89	20.24	1	1	26.61	29.72	not confirmed, different phenotype	MSD::6::13::13_NOCODAZOLE	-2.56	-0.37418758495990206, -4.746318745107297
ADD::11::13::10	1			18.07			3	1	2.76	4.96	not confirmed, no phenotype	ADD::11::13::10_	-1.78	-3.366014701453263, -0.18637716815760622
MSD::3::15::6	0	1	1	10.13	20.98	20.28	2	2	16.87	17.95	not confirmed, different phenotype	MSD::3::15::6_SARMENTOGENIN	-1.57	-1.3559998532717017, -1.7790734334844616
ADD::12::1::12	1			27.76			2	2	46.60	53.25	not confirmed, different phenotype	ADD::12::1::12_	-1.34	-2.743420584598591, 0.07168691094914664
CBN::50::9::7	1			27.55			2	2	68.17	68.79	not confirmed, different phenotype	CBN::50::9::7_	-1.31	-1.4245152364490536, -1.1924631027895674
MSD::5::12::9	0	1	1	64.03	30.99	40.65	2	2	76.00	65.64	not confirmed, different phenotype	MSD::5::12::9_STROPHANTHIDINIC ACID L	-1.23	-1.0837550865746313, -1.3738616802502963
MSD::2::5::5	0	1	0	64.31	37.76	46.33	2	2	21.62	22.37	not confirmed, different phenotype	MSD::2::5::5_GITOXIN	-1.12	-0.9175389814920875, -1.313394618905151
CBN::50::3::20	1			19.18			3	3	36.04	37.00	not confirmed, different phenotype	CBN::50::3::20_	-1.10	-1.0388982368006283, -1.1578271576036379
KBI::2::5::8	1	2		22.65	14.06		3	3	25.71	21.58	not confirmed, different phenotype	KBI::2::5::8_SP-600125	-0.96	-0.7965436777807849, -1.1205298447184397
ADD::31::6::21	1			18.51			1	1	25.70	25.39	not confirmed, different phenotype	ADD::31::6::21_	-0.87	-0.9666891600849798, -0.781812072954575
KBI::2::3::15	1	0		30.21	17.96		3	2	19.59	24.80	not confirmed, different phenotype	KBI::2::3::15_DRB	-0.80	-0.33773467427977694, -1.2697905060555699
ADD::30::13::6	1			16.70			3	2	4.31	3.56	not confirmed, no phenotype	ADD::30::13::6_	-0.72	-0.722214983753905, -0.722030781735325
KBI::2::5::20	1	0		26.09	18.60		3	3	24.41	27.43	not confirmed, different phenotype	KBI::2::5::20_Roscovitine	-0.68	-0.5723221802111585, -0.7973482269503969
CBN::20::4::10	1			23.04			3	3	31.33	35.62	not confirmed, different phenotype	CBN::20::4::10_	-0.67	-0.46397004137849385, -0.8720791355832409
FAN::1::4::13	1			17.80			1	4	6.86	3.59	not confirmed, no phenotype	FAN::1::4::13_	-0.61	-1.1213008841719296, -0.10201382341266642
MSD::7::16::5	0	1	1	20.32	22.52	24.32	3	3	21.79	21.73	not confirmed, different phenotype	MSD::7::16::5_LYCORINE	-0.53	-0.3719997961144003, -0.6795151777473927
PCP::1::12::15	1			19.03			1	1	24.68	29.22	not confirmed, different phenotype	PCP::1::12::15_	-0.49	-0.6882388024293611, -0.2830114744950728
ADD::52::1::19	1			23.78			2	1	27.90	27.07	not confirmed, different phenotype	ADD::52::1::19_	-0.48	-0.5120299280471893, -0.44985099254973454
MSD::1::1::20	0	1	0	46.50	30.03	42.78	1	1	25.73	27.07	not confirmed, different phenotype	MSD::1::1::20_SAPPANONE A 7-METHYL	-0.43	-0.5623831019247076, -0.30226412895099186
CBN::34::1::18	1			16.03			1	1	20.11	20.48	not confirmed, different phenotype	CBN::34::1::18_	-0.41	-0.45186189176290664, -0.3748654493187984
ADD::44::16::12	1			16.64			1	1	8.34	2.78	not confirmed, different/no phenotype	ADD::44::16::12_	-0.40	-0.3029648280256619, -0.4985015993419409
CBN::31::14::21	1			23.55			3	3	39.61	38.05	not confirmed, different phenotype	CBN::31::14::21_	-0.39	-0.2800134752059548, -0.4910541281373139
ADD::44::16::20	1			15.32			1	1	3.49	5.45	not confirmed, no phenotype	ADD::44::16::20_	-0.32	-0.26015048116677053, -0.3841938632206639
MSD::3::11::8	0	1	1	36.45	42.63	49.01	3	3	49.10	48.42	not confirmed, different phenotype	MSD::3::11::8_EMETINE	-0.26	-0.5445731058119532, 0.027998216452927528
MSD::1::10::15	0	1	1	18.61	21.95	26.76	2	2	26.65	27.56	not confirmed, different phenotype	MSD::1::10::15_GINKGETIN; K salt	-0.24	-0.1468009833110178, -0.3320209707691891
KBI::2::7::15	1	1		15.20	11.84		4	0	3.94	7.10	not confirmed, no phenotype	KBI::2::7::15_HA-1004	-0.24	-0.27586552927502606, -0.1965972244370510
ADD::33::4::19	1			16.74			3	3	5.79	2.96	not confirmed, no phenotype	ADD::33::4::19_	-0.21	-0.12933787432109725, -0.2877077350333048
ADD::31::12::17	1			15.87			3	3	5.00	4.76	not confirmed, no phenotype	ADD::31::12::17_	-0.21	-0.22195854875855367, -0.1906404964415735
MSD::3::8::11	0	0	1	22.79	24.73	25.88	3	2	13.66	11.49	not confirmed, different phenotype	MSD::3::8::11_GEDUNIN	-0.19	-0.3651272450528481, -0.0135638753991575
CBN::51::12::7	1			21.09			3	0	10.86	6.38	not confirmed, different/no phenotype	CBN::51::12::7_	-0.18	-1.6694978905352797, 1.308345367373247
KBI::2::1::20	1	0		25.45	16.09		1	1	4.68	11.64	not confirmed, different/no phenotype	KBI::2::1::20_SU-4312	-0.17	0.16921996923921542, -0.5122637064737066
KBI::2::2::15	1	3		26.45	20.24		1	1	42.48	41.35	not confirmed, different phenotype	KBI::2::2::15_Nocodazole	-0.12	0.2120777164421151, -0.4569801890625089
CBN::52::16::7	1			19.24			2	1	5.34	2.91	not confirmed, no phenotype	CBN::52::16::7_	-0.11	-0.10348330948275729, -0.1261771772955175
CBN::39::8::5	1			17.80			4	4	1.40	4.44	not confirmed, no phenotype	CBN::39::8::5_	-0.09	-0.17001065969325452, -0.0134615219474351
CBN::58::7::20	1			18.70			2	1	1.91	3.98	not confirmed, no phenotype	CBN::58::7::20_	-0.08	0.036455889280140395, -0.2006782318840912
CBN::13::12::7	1			19.69			3	0	7.01	5.21	not confirmed, no phenotype	CBN::13::12::7_	-0.06	0.342344897726335, -0.46917515449049174
MSD::2::13::5	0	1	1	21.04	24.56	12.82	1	1	32.48	32.59	not confirmed, different phenotype	MSD::2::13::5_ROTENONE	-0.05	-0.035556792077513916, -0.060189375806219
MSD::5::9::20	1	1	1	7.10	11.63	24.45	3	3	5.41	2.93	not confirmed, no phenotype	MSD::5::9::20_CANDESARTAN CILEXTIL	-0.03	-0.05867075900770873, -8.378789674519441E
KBI::2::15::10	1	2		18.40	15.54		1	1	29.34	31.22	not confirmed, different phenotype	KBI::2::15::10_Mycophenolic acid	-0.03	0.11917554897237918, -0.17352140339605462
MSD::2::12::18	1	1	1	17.19	22.94	21.26	1	1	23.01	22.28	not confirmed, different phenotype	MSD::2::12::18_MEBENDAZOLE	-0.01	-0.09747793647673533, 0.0837487379224551
KBI::2::2::11	1	0		31.18	33.67		3	3	26.39	28.45	not confirmed, different phenotype	KBI::2::2::11_Lycorine	0.00	0.15969358950814805, -0.15541768004412074
KBI::2::11::16	1	2		15.16	2.29		4	4	2.71	1.49	not confirmed, no phenotype	KBI::2::11::16_AG-370	0.02	0.23087270219883432, -0.1884518179527316
CBN::19::6::6	1			20.34			3	3	10.78	4.18	not confirmed, different/no phenotype	CBN::19::6::6_	0.03	0.4030585459229317, -0.3471277768309683
FAN::1::10::8	1			20.58			4	0	3.50	5.82	not confirmed, no phenotype	FAN::1::10::8_	0.04	0.2953117746021213, -0.21755337339504877
CBN::11::14::12	1			22.52			1	0	3.40	5.50	not confirmed, no phenotype	CBN::11::14::12_	0.14	0.36258657871479194, -0.0735862627437650
PCP::3::13::22	1			27.97			3	3	29.08	31.27	not confirmed, different phenotype	PCP::3::13::22_	0.17	0.6283354007080343, -0.28219821750418667
KBI::2::7::13	2	1		11.81	19.90		4	4	9.39	10.27	not confirmed, different phenotype	KBI::2::7::13_HA-1077	0.19	0.20302354389770289, 0.17720911104341686
CBN::11::7::22	1			15.89			4	1	2.39	5.68	not confirmed, no phenotype	CBN::11::7::22_	0.30	0.3643153380335422, 0.2276187794770365
KBI::2::3::9	1	0		18.70	16.92		1	1	37.20	36.37	not confirmed, different phenotype	KBI::2::3::9_Diphenyleneiodonium	0.37	0.39247123787302746, 0.3529065194866148
MSD::5::13::7	0	0	1	61.24	45.98	41.75	2	2	35.15	35.86	not confirmed, different phenotype	MSD::5::13::7_HEXACHLOROPHENE	0.77	0.8039801741769235, 0.7264997390955265
MSD::5::6::14	4	0	1	22.40	18.46	21.25	1	1	28.26	28.82	not confirmed, different phenotype	MSD::5::6::14_HOMIDIUM BROMIDE	8.83	8.086553265868083, 9.564184438026903
FAN::1::2::19	1			107.83			2	2	127.31	110.43	not confirmed, different phenotype	FAN::1::2::19_	112.92	109.87213072589311, 115.96005974904027
ADD::10::13::13	1			16.33			0	0	23.37	16.64	confirmed twice, behaves like TGF R inhibitor	ADD::10::13::13_	-1.74	-0.6711956371202595, -2.8110370955300583
CBN::4::16::7	1			26.83			0	0	55.25	58.05	confirmed twice, behaves like TGF R inhibitor	CBN::4::16::7_	-1.67	-1.947810251973319, -1.3977086952366948
MSD::1::14::15	0	1	1	45.41	33.78	34.48	0	0	62.69	62.17	confirmed twice, behaves like TGF R inhibitor	MSD::1::14::15_STROPHANTHIDIN	-1.45	-1.2868100080284357, -1.6198797490104626
CBN::4::14::9	1			26.68			0	0	47.69	45.44	confirmed twice, behaves like TGF R inhibitor	CBN::4::14::9_	-1.45	-1.1882686886935754, -1.711145540601775
CBN::42::7::9	1			16.96			0	0	14.44	9.49	confirmed twice, behaves like TGF R inhibitor	CBN::42::7::9_	-1.36	-1.8199191829462467, -0.8977096531413443
CBN::42::7::10	1			16.42			0	0	12.75	10.92	confirmed twice, behaves like TGF R inhibitor	CBN::42::7::10_	-1.14	-1.4362529763633394, -0.8487229350909088
MSD::6::8::13	1	1	1	24.38	29.26	31.69	0	0	40.20	45.30	confirmed twice, p-Smad2 unclear	MSD::6::8::13_CYCLOHEXIMIDE	-0.98	-1.9988411689301582, 0.0385430699087272
CBN::33::9::12	1			16.98			0	0	18.94	17.20	confirmed twice, p-Smad2 unclear	CBN::33::9::12_	-0.70	-1.5296043329141569, 0.13910104936069234
MSD::4::2::7	1	1	1	3.47	6.62	15.60	0	0	12.22	10.11	confirmed twice, p-Smad2 unclear	MSD::4::2::7_S;S;S;-TRIBUTYLPHOSPHOF	-0.64	-1.1350750634063829, -0.14094613601975656
CBN::49::1::14	1			17.60			0	0	14.49	28.26	confirmed twice, p-Smad2 unclear	CBN::49::1::14_	-0.53	-0.5645114488270677, -0.5039374198552067
ADD::41::8::22	1			26.65			0	0	10.09	8.76	confirmed twice, selected compound	**ADD::41::8::22_**	-0.43	-0.2583904775288627, -0.6070807132300396
MSD::1::7::21	1	1	1	14.16	7.68	22.50	0	0	7.56	8.78	confirmed twice, selected compound	**MSD::1::7::21_LANOSTEROL**	-0.41	-0.6011210252376602, -0.22708726459320547
MSD::7::16::15	0	1	0	23.35	32.10	25.19	0	0	44.63	56.29	confirmed twice, selected compound	**MSD::7::16::15_BUMETANIDE**	-0.39	-0.18158929330793583, -0.6044261329241579
ADD::14::13::21	1			51.63			0	0	72.76	65.98	confirmed twice, selected compound	**ADD::14::13::21_**	-0.22	-0.1437031528255689, -0.29343485254256874
ADD::12::13::17	1			15.69			0	0	20.93	16.05	confirmed twice, selected compound	**ADD::12::13::17_**	-0.19	-0.09935174616328787, -0.2795300855234429
CBN::36::16::5	1			23.86			0	0	9.11	9.64	confirmed twice, selected compound	**CBN::36::16::5_**	-0.14	0.22880675568911732, -0.5186069988928058
ADD::12::11::17	1			26.72			0	0	20.80	21.63	confirmed twice, selected compound	**ADD::12::11::17_**	-0.09	0.010064484409359352, -0.1891869362244177
MSD::3::2::17	1	0	0	15.81	71.65	85.88	0	0	15.12	10.23	confirmed twice, selected compound	**MSD::3::2::17_ESTRADIOL VALERATE**	0.02	-0.3533255594642424, 0.399165622254421
CPW::1::6::8	1	1	1	16.11	14.13	17.14	0	0	12.50	8.92	confirmed twice, conflict of interest	CPW::1::6::8_	0.05	0.19233536322404818, -0.09812751105016242
ADD::7::16::20	1			15.88			0	0	13.53	8.71	confirmed twice, selected compound	**ADD::7::16::20_**	0.07	0.23661664635064406, -0.09732227712951863
CBN::49::3::19	1			15.05			0	0	8.63	9.60	confirmed twice, selected compound	**CBN::49::3::19_**	0.12	0.5065049548831972, -0.25836967312514536
KBI::2::9::7	1	0		22.79	21.84		0	0	14.33	7.82	confirmed twice, selected compound	**KBI::2::9::7_24(S)-Hydroxycholesterol**	0.23	0.5837814887955454, -0.11992157901464458
CBN::38::7::21	1			15.75			0	0	16.36	20.30	confirmed twice, selected compound	**CBN::38::7::21_**	0.27	0.4603150181297458, 0.07648579367281769
MSD::6::6::13	1	1	0	18.12	30.74	23.16	0	0	37.51	42.81	confirmed twice, selected compound	**MSD::6::6::13_ACIVICIN**	0.35	0.8921780493021418, -0.1850829007952017
MSD::1::7::8	1	1	1	4.79	10.89	23.46	0	0	16.29	14.48	confirmed twice, selected compound	**MSD::1::7::8_4-NONYLPHENOL**	1.26	-0.1482811430329249, 2.6618313145993593
KBI::2::5::6	1	1		44.64	54.81		0	3	35.91	35.37	confirmed once, behaves like TGF R inhibitor	KBI::2::5::6_SB-431542	-6.37	-6.365431098682444, -6.37775103326747
MSD::3::6::14	0	1	0	63.21	33.46	48.18	2	0	26.18	34.75	confirmed once, behaves like TGF R inhibitor	MSD::3::6::14_GITOXIGENIN DIACETATE	-1.57	-1.507578273992867, -1.6330795192687924
ADD::34::6::19	1			15.26			3	0	19.74	16.97	confirmed once, p-Smad2 unclear	ADD::34::6::19_	-0.97	-1.1522761124064407, -0.7793317952546721
CBN::4::6::9	1			16.17			3	0	26.70	33.29	confirmed once, p-Smad2 unclear	CBN::4::6::9_	-0.83	-0.8831453431144489, -0.7812544675925127
MSD::6::5::21	1	1	1	20.40	22.55	24.63	2	0	27.74	31.76	confirmed once, p-Smad2 unclear	MSD::6::5::21_THIOGUANINE	-0.54	-0.3674102675703122, -0.7208564340093442
KBI::2::11::15	1	1		29.04	14.78		3	0	8.65	10.97	confirmed once, p-Smad2 unclear	KBI::2::11::15_N9-Isopropylolomoucine	-0.42	-0.5319431297111858, -0.3163681464301107
MSD::1::5::8	0	1	1	21.88	21.10	24.66	3	0	34.22	39.54	confirmed once, p-Smad2 unclear	MSD::1::5::8_CRINAMINE	-0.37	-0.42952737298397703, -0.3149422972802915
CBN::42::1::10	1			17.36			0	0	10.92	7.25	confirmed once, selected compound	**CBN::42::1::10_**	-0.17	-0.027519232753788377, -0.321578942882403
MSD::2::7::12	1	1	1	12.29	10.94	17.21	0	0	7.67	5.68	confirmed once, selected compound	**MSD::2::7::12_TOTARALOLAL**	-0.11	-0.12263477482592394, -0.0892599786889273
ADD::34::16::8	1			15.08			0	3	8.10	2.17	confirmed once, compound not selected	ADD::34::16::8_	-0.06	-0.6411815676514552, 0.5124465535269891

- confirmed twice, selected compound
- confirmed twice, conflict of interest
- confirmed twice, behaves like TGF R inhibitor
- confirmed twice, p-Smad2 unclear
- confirmed once, selected compound
- confirmed once, compound not selected
- confirmed once, behaves like TGF R inhibitor
- confirmed once, p-Smad2 unclear
- not confirmed, twice in EMT cluster
- not confirmed, different phenotype
- not confirmed, different/no phenotype
- not confirmed, no phenotype

selected

not selected

p-Smad2 influence

Figure S5. The 93 primary hits. The table lists all 93 primary hits with their library code names (1st column), recovery in up to three independent repeats of the primary screen (A, B, C, where the number indicates the cluster after k-means clustering and 1 was the EMT-specific cluster, 2nd to 4th columns), phenotypic strength as Euclidian length of the parameter profile in each or the repeats (where > 15 was considered to be a hit, 5th to 7th columns), recovery in the two verification screens (where the number indicates the cluster after k-means clustering and 0 was the EMT-specific cluster, 8th and 9th column) and corresponding phenotypic strengths (where > 7.5 was considered to be a hit, 10th and 11th column) and description of the hit verification (12th column). The colour coding of the latter (12th) column is explained below the table. The same 93 compounds with their code names are listed again (13th column) and the colour code explained below the table indicates behavior of the compounds in the secondary screen where compounds influencing p-Smad2 nuclear localisation were discriminated. The mean scores (14th column) and ranges (15th and 16th columns) of the cytoplasmic to nuclear ratios quantified for each compound are finally shown.

Supplementary Figure 6 Carthy et al

A

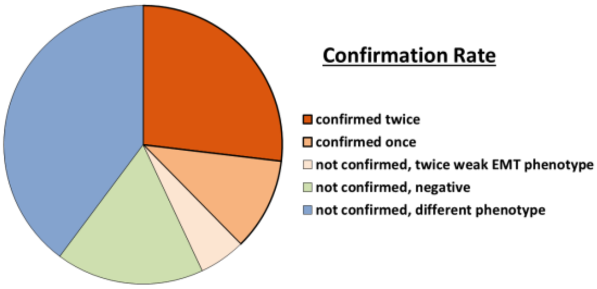

B

Scatter plot of Fibronectin and p-Smad2 assay for 35 confirmed hits

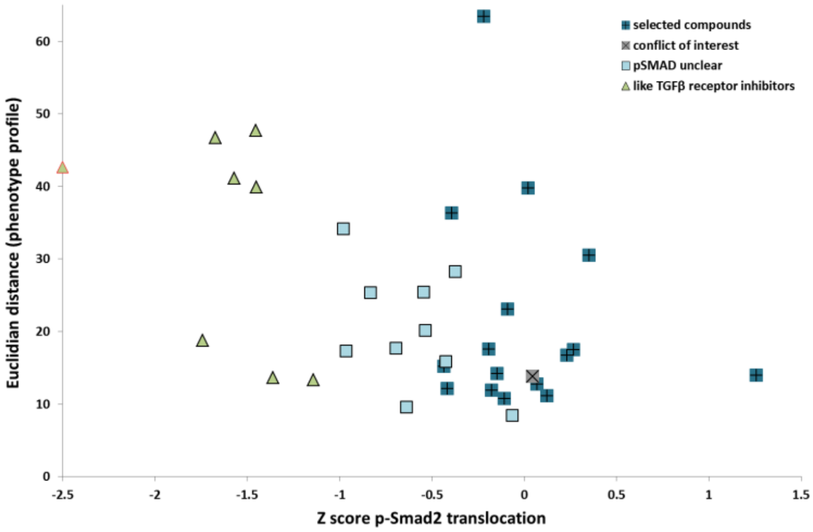

C

Molecule	library code ::library plate number::plateRow::plate Column	A::prim Screen	B::prim Screen	A::prim Screen (vector-norm)	B::prim Screen (vector-norm)	A::verif Screen(clusterVerifScreen)	B::verif Screen(clusterVerifScreen)	A::verif Screen(vector-norm)(clusterVerifScreen)	B::verif Screen(vector-norm)(clusterVerifScreen)	hitVerification(clusterVerif Screen)	CytNuc_MEAN_CircRingAvgIntenRatioCh2.poc.zscore (Mean)	CytNuc_MEAN_CircRingAvgIntenRatioCh2.poc.zscore (Concatenate)
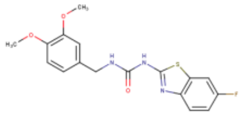	ADD::34::16::8	1	?	15.079	?	0	3	8.103	2.169	primaryHit_confirmedOnce	-0.064	-0.6411815676514552, 0.5124465535269891
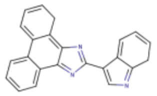	CBN::49::1::14	1	?	17.602	?	0	0	14.488	28.259	primaryHit_confirmedTwice	-0.534	-0.5645114488270677, -0.5039374198552067
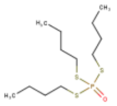	MSD::4::2::7	1	1	3.47	6.616	0	0	12.216	10.105	primaryHit_confirmedTwice	-0.638	-1.1350750634063829, -0.14094613601975656
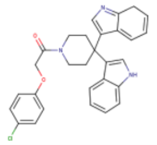	CBN::42::7::9	1	?	16.961	?	0	0	14.44	9.494	primaryHit_confirmedTwice	-1.359	-1.8199191829462467, -0.8977096531413443
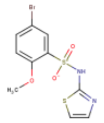	ADD::10::13::13	1	?	16.326	?	0	0	23.371	16.644	primaryHit_confirmedTwice	-1.741	-0.6711956371202995, -2.8110370955300583
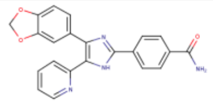	KBI::2::5::6	1	1	44.636	54.807	0	3	35.91	35.367	primaryHit_confirmedOnce	-6.372	-6.365431098682444, -6.37775103326747

Figure S6. Confimation rate and putative useful compounds. (**A**) A pie chart indicating the relative number of compounds according to their verification category. The colour coding of the pie chart corresponds to the colours of the table in Fig. S5, 12th column, and the small table below. The percent of compounds (out of 93 total) that corresponds to each colour is: 27% of hits were confirmed twice (dark orange), 11% of hits were confirmed once (orange), 5% of hits were not confirmed, but were grouped twice in the EMT cluster (light orange), 17% of hits were not confirmed due to absence of phenotype (green), and 40% of hits were not confirmed due to different phenotype (blue). (**B**) Scatter plot of the confirmed 35 hits showing the phenotypic strength, expressed as Euclidian length of the parameter profile in relation to the p-Smad2 translocation. Compounds that inhibited both fibronectin and p-Smad2 translocation appear on the left side of the plot (green triangles). These compounds are interpreted as acting similar to TGF-β receptor kinase inhibitors. Compounds that inhibited fibronectin without affecting p-Smad2 translocation (scatter around zero) are the selected compounds (blue squares with cross). Among them, one compound presented a proprietary conflict of interest (grey square with an x). Unclear compounds cluster in the center of the plot (light blue squares). (**C**) Table of specific compounds that were not selected as hits. The table lists the compound structure and additional features similar to those listed in Fig. S5.

Supplementary Figure 7 Carthy et al

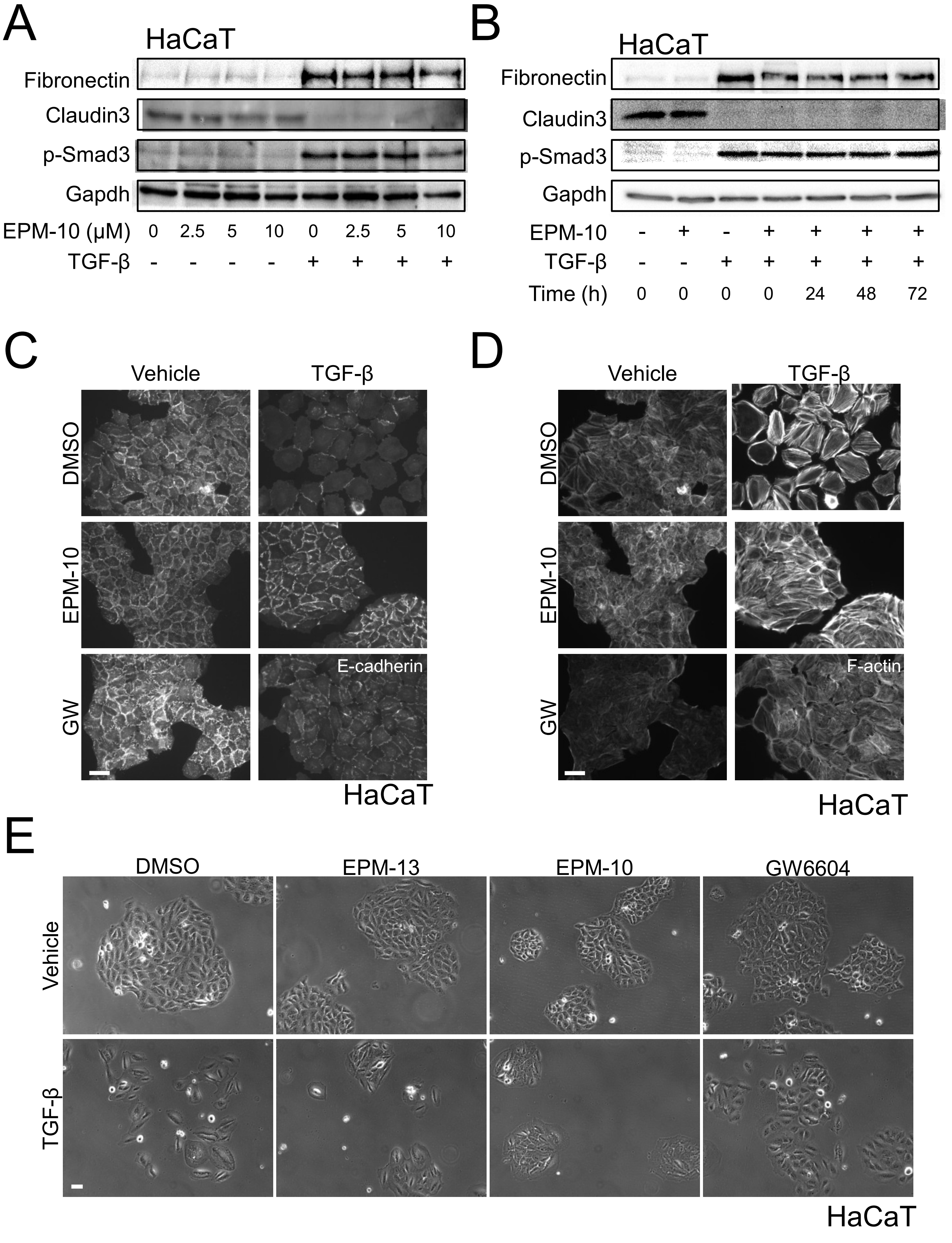

Figure S7. MET induced by EPM-10 and EPM-13. (**A**) Protein expression analysis in total cell lysates from HaCaT cells stimulated (+) or not (-) with 5 ng/ml TGF-β for a total of 96 h and co-treated with DMSO (0 μM) or specific EPM-10 compound at the indicated concentrations. Immunoblot for the indicated proteins and for Gapdh, which serves as protein loading control. (**B**) Protein expression analysis in total cell lysates from HaCaT cells (+) stimulated or (-) not with 5 ng/ml TGF-β for a total of 96 h and co-treated with DMSO (0 μM) or specific EPM-10 compound at 10 μM concentration at the indicated time points following TGF-β stimulation. Immunoblot for the indicated proteins and for Gapdh, which serves as protein loading control. (**C**) Immunofluorescence microscopy of HaCaT cells for the epithelial protein E-cadherin in cells stimulated with vehicle or 5 ng/ml TGF-β for 96 h in the presence of DMSO or 10 μM of EPM-10 or 3.3 μM GW6604 (GW). (**D**) Direct fluorescence microscopy of HaCaT cells for actin microfilaments in cells treated as in panel C with the indicated compounds. (**E**) Phase contrast microscopy of HaCaT cells treated as in panel C with the indicated compounds. White bars indicate 10 μm.

Supplementary Figure 8 Carthy et al

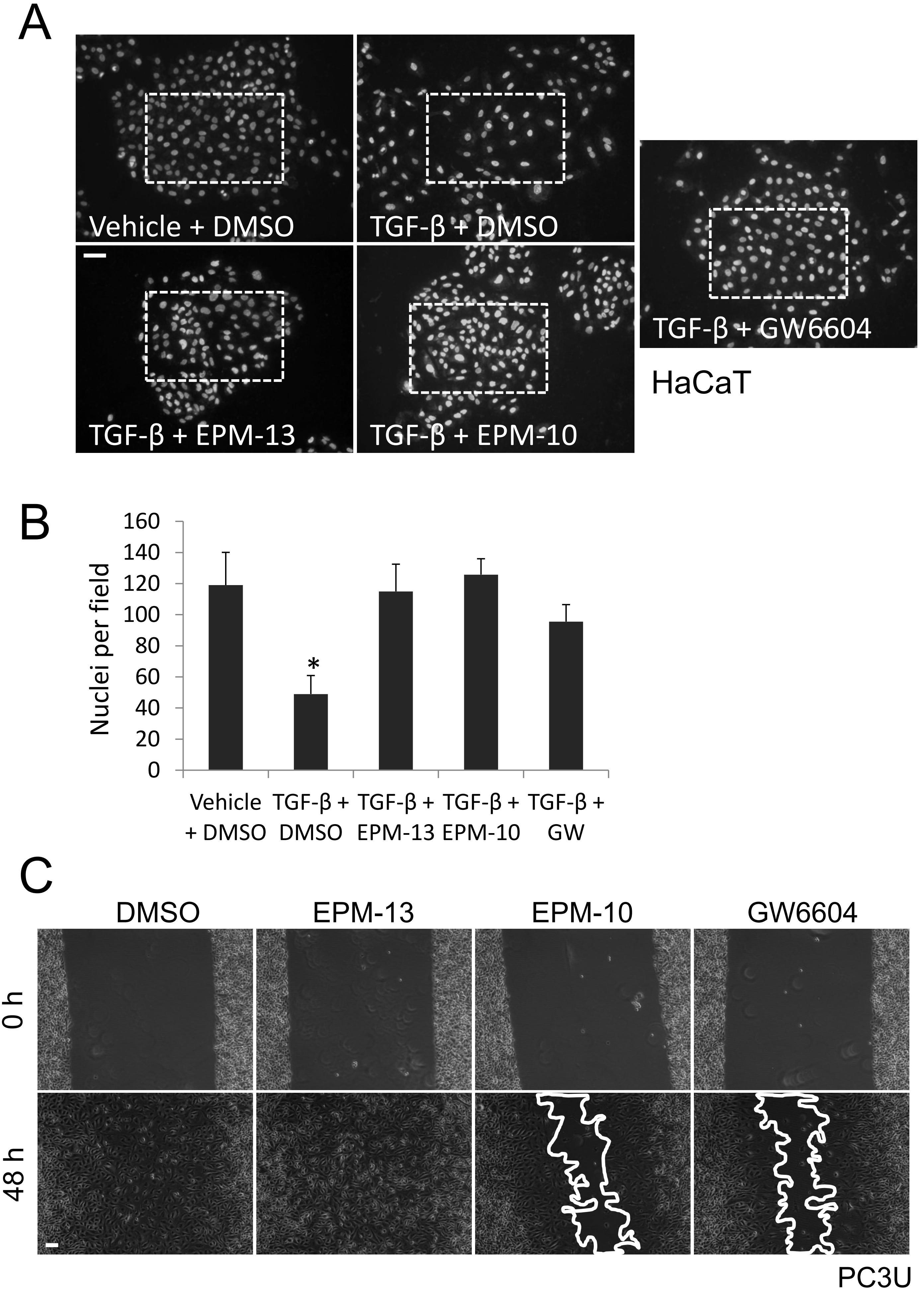

Figure S8. Compact cell-cell adhesion induced by EPM-10 and EPM-13. (**A**) DAPI staining of HaCaT cell nuclei stimulated with vehicle or 5 ng/ml TGF-β for 96 h in the presence of DMSO or 10 μM of EPM-10, EPM-13 or 3.3 μM GW6604 (GW). Dotted frames indicate the cells that were counted to generate the data graphed in panel B. (**B**) Graph of cell nuclei counted from the micrographs of panel A. Average values derived from 5 frames with standard errors are plotted and a star indicates significance at $p<0.05$. (**C**) Two dimensional wound healing assay of prostate cancer PC3U cells migrating for 48 h after the scratch and treated with compounds EPM-10 and EPM-13 at 10 μM or 3.3 μM GW6604, at the start of the time period. Phase contrast micrographs are shown and white lines indicates the open area left at the end of the 48 h period. White bars indicate 10 μm.

Supplementary Figure 9 Carthy et al

A

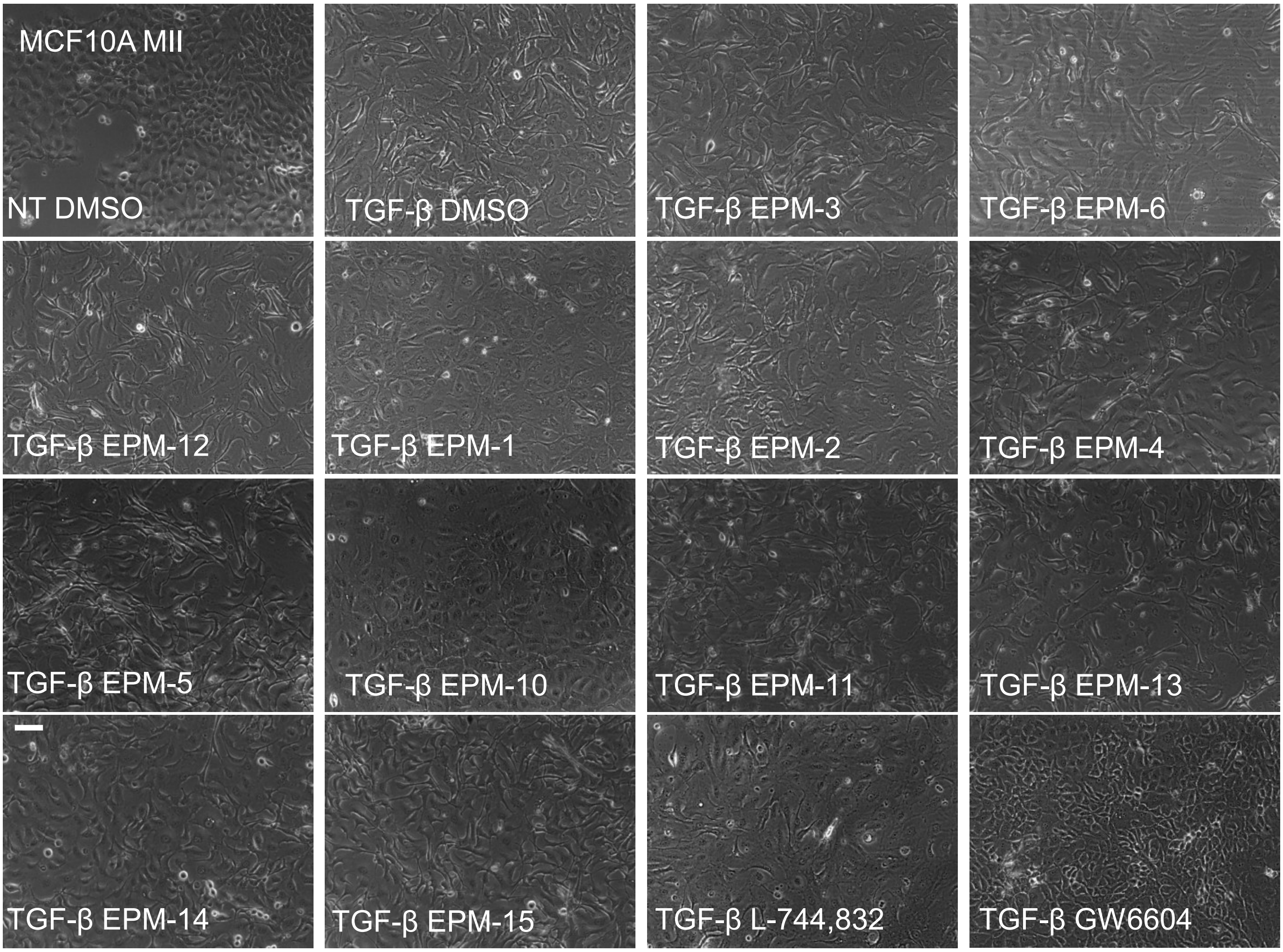

B

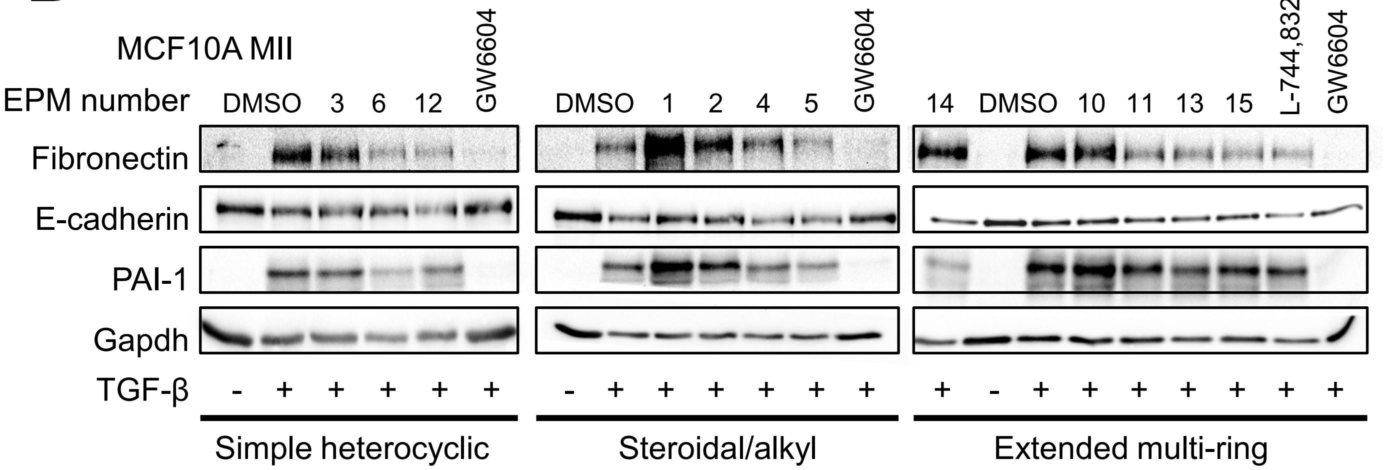

Figure S9. Analysis of 13 selected compounds in breast cancer cells. (**A**) Phase contrast microscopy of MCF10A-MII cells stimulated (+) or not (-) with 5 ng/ml TGF-β for a total of 96 h and co-treated with DMSO or specific EPM compounds at 10 μM final concentration. A white bar indicates 10 μm. (**B**) Protein expression analysis in total cell lysates from breast cancer MCF10A-MII cells stimulated (+) or not (-) with 5 ng/ml TGF-β for a total of 96 h and co-treated with DMSO or specific EPM compounds at 10 μM final concentration. Immunoblot for the indicated proteins and for Gapdh, which serves as protein loading control.

Supplementary Figure 10 Carthy et al

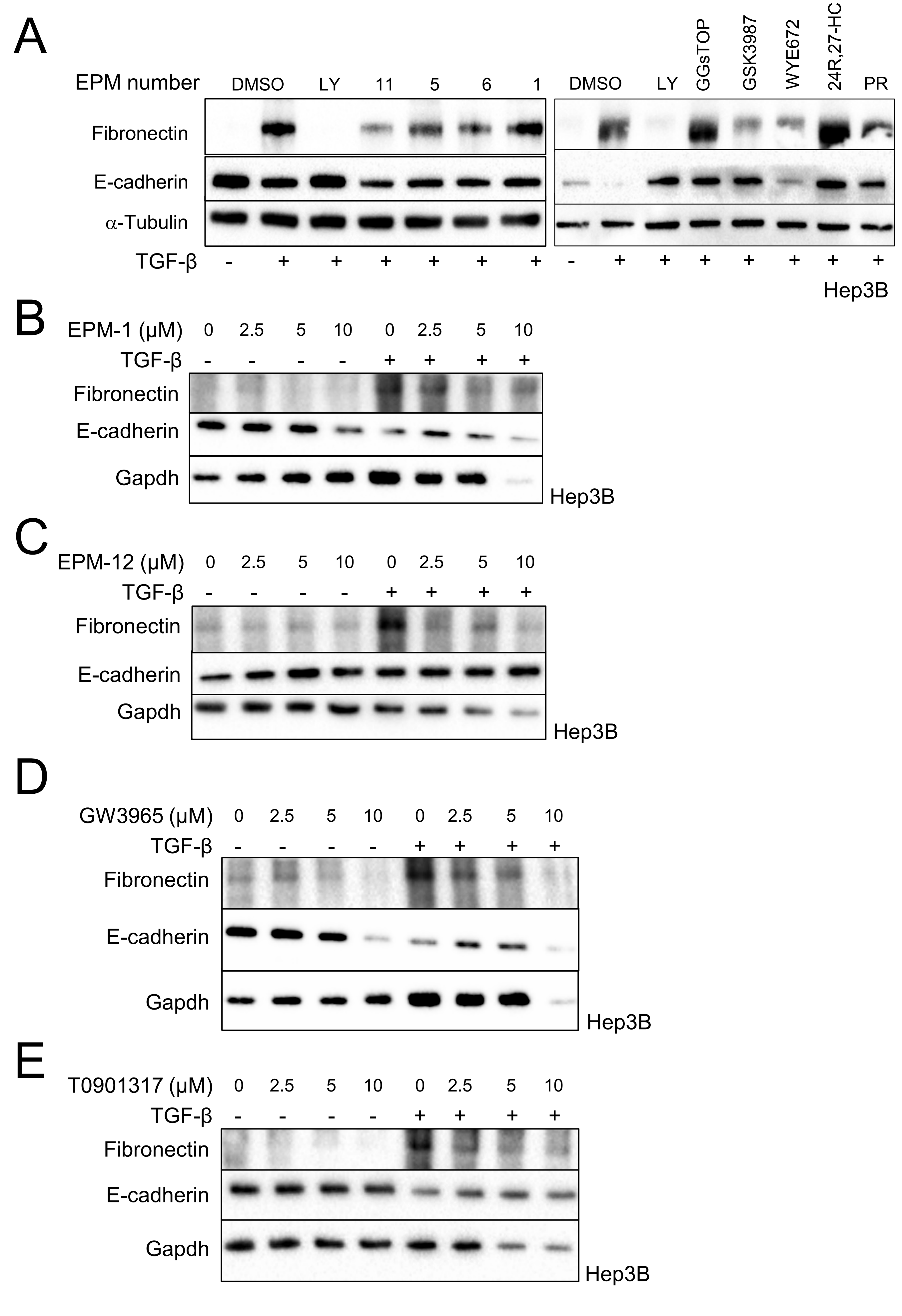

Figure S10. Analysis of selected compounds in HCC cells. (**A**) Protein expression analysis in total cell lysates from Hep3B HCC cells stimulated (+) or not (-) with 5 ng/ml TGF-β for a total of 72 h (left panel) or 48 h (right panel) and co-treated with DMSO or specific EPM compounds at 100 nM (left panel) or 1 μM (right panel) final concentration. LY is the TGF-β type I receptor kinase inhibitor LY2109761. Immunoblot for the indicated proteins and for α-tubulin, which serves as protein loading control. (**B-E**) Protein expression analysis in total cell lysates from Hep3B cells stimulated (+) or not (-) with 5 ng/ml TGF-β for a total of 72 h and co-treated with DMSO (0 μM) or specific EPM-1 (B), EPM-12 (C), GW3965 (D) and T0901317 (E) compounds at the indicated concentrations. Immunoblot for the indicated proteins and for Gapdh, which serves as protein loading control.

Supplementary Figure 11 Carthy et al

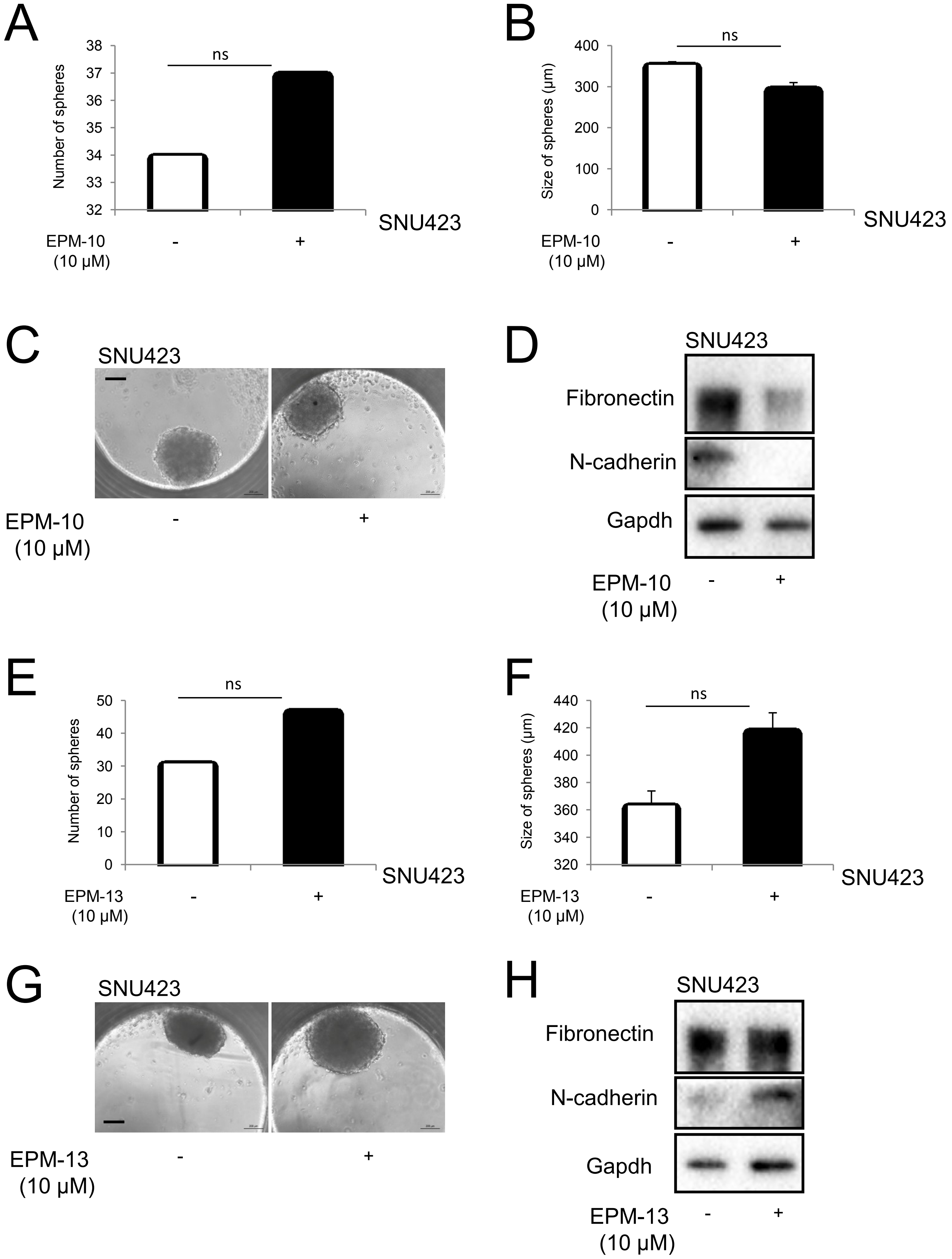

Figure S11. Impact of selected compounds on HCC hepatosphere formation and MET. (**A, B, E, F**) Number (A, E) and size (B, F) of SNU423 hepatospheres grown in hanging drops using Insphero assays in the presence of control, DMSO or 10 µM EPM-10 (A, B) or 10 µM EPM-13 (E, F). The data are expressed as bar graphs of average determinations with corresponding standard errors from triplicate determinations. Absence of significant difference at $p<0.05$ is indicated (ns). (**C, G**) Representative phase contrast images of hepatospheres grown in hanging drops using Insphero assays in the presence of control, DMSO or 10 µM EPM-10 (C) or 10 µM EPM-13 (G). Bars indicate 50 µm. (**D, H**) Protein expression analysis in the hepatospheres treated with DMSO or 10 µM EPM-10 (D) or 10 µM EPM-13 (H) under the same conditions as in the other panels. Immunoblots for the indicated proteins and Gapdh, the protein loading control, are shown.

Supplementary Figure 12 Carthy et al

A

Target	Compound	Pharmacology
LXR	24, 25 Epoxycholesterol	Agonist
LXR	T0901317	Agonist
LXR	GW3965	Agonist
LXR	Tularik antagonist (cmpd 54)	Antagonist
LXR	GSK antagonist (GSK 2033)	Antagonist
ER/PR	Estradiol	Agonist
ER/PR	DES	Agonist
ER/PR	Tamoxifen	Antagonist
ER/PR	Progesterone	Agonist
ER/PR	Mifepristone (RU486)	Antagonist
PXR	PCN	Agonist
PXR	SR12813	Agonist
CAR	TCOBOP	Agonist
CAR	CITCO	Agonist
GMP synthase inhibitor	Decoyinine	Antagonist
Glutamine analog	Azaserine	Antagonist
Glutamine analog	DON	Antagonist

C

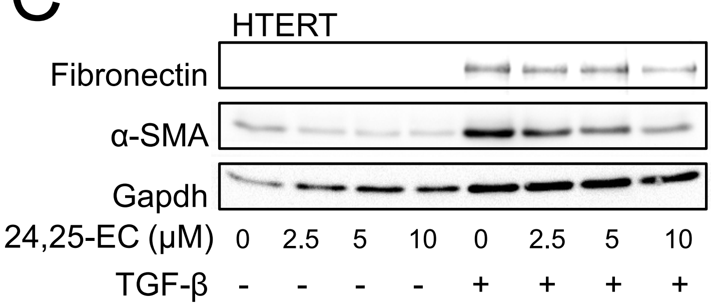

D

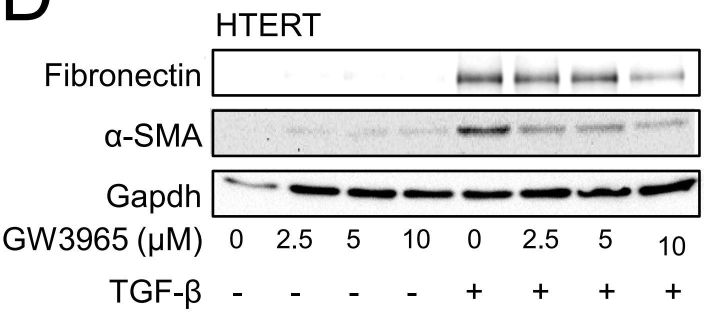

E

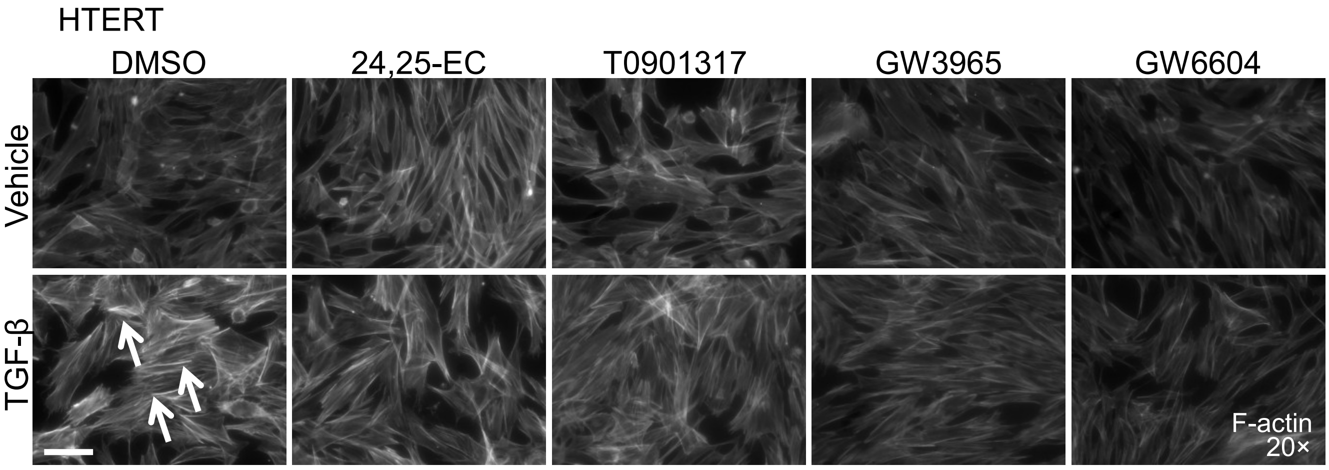

Figure S12. LXR agonists inhibit myofibroblast differentiation. (**A**) A list of nuclear receptor agonists and antagonists with their pharmacological specificity and a short list of additional compounds that block glutamine synthesis. (**B, C**) Protein expression analysis in total cell lysates from HTERT cells stimulated (+) or not (-) with 5 ng/ml TGF-β for a total of 72 h and co-treated with DMSO (0 μM) or specific 24, 25 epoxycholesterol (EC) (B) and GW3965 (C) compounds at the indicated concentrations. Immunoblot for the indicated proteins and for Gapdh, which serves as protein loading control. (**D**) Direct fluorescence microscopy of HTERT cells for actin microfilaments in cells stimulated with vehicle or 5 ng/ml TGF-β for 72 h in the presence of DMSO or 10 μM of the indicated compounds or 3.3 μM of GW6604. White arrows mark TGF-β-inducible α-SMA microfilaments and a white bar indicates 10 μm.

Supplementary Figure 13 Carthy et al

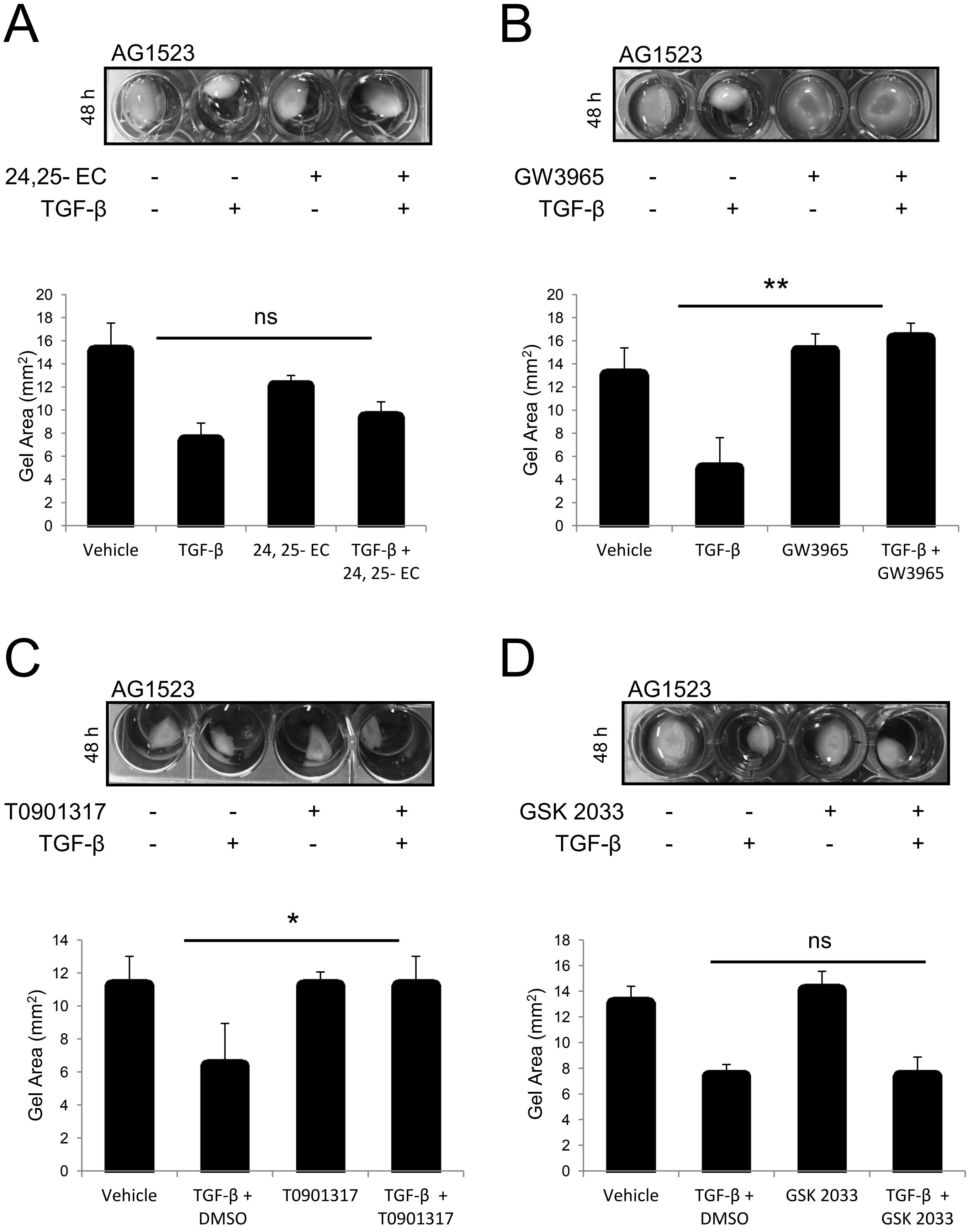

Figure S13. Impact of LXR agonists and antagonists on myofibroblast contractility. Collagen gel contraction assays performed on AG1523 cells stimulated (+) or not (-) with 5 ng/ml TGF-β for 48h in the presence (+) or absence (-, DMSO control) of LXR agonists 24, 25-epoxycholesterol (24, 25-EC) (**A**), GW3965 (**B**), T0901317 (**C**) or LXR antagonist GSK 2033 (**D**). A representative image and corresponding quantification of contracted gels graphed as average of 5 repeats with associated standard deviation. Stars indicate statistically significant differences at $p<0.05$ (*) or $p<0.01$ (**) or lack of significance at $p<0.05$ (ns). Note that the data of panel C using T0901317, recording cell contractility at 48 h, represent an independent biological repeat of the experiment shown in Fig. 6D, which recorded cell contractility at 72 h, demonstrating robust reproducibility at two independent time points.

Supplementary Figure 14 Carthy et al

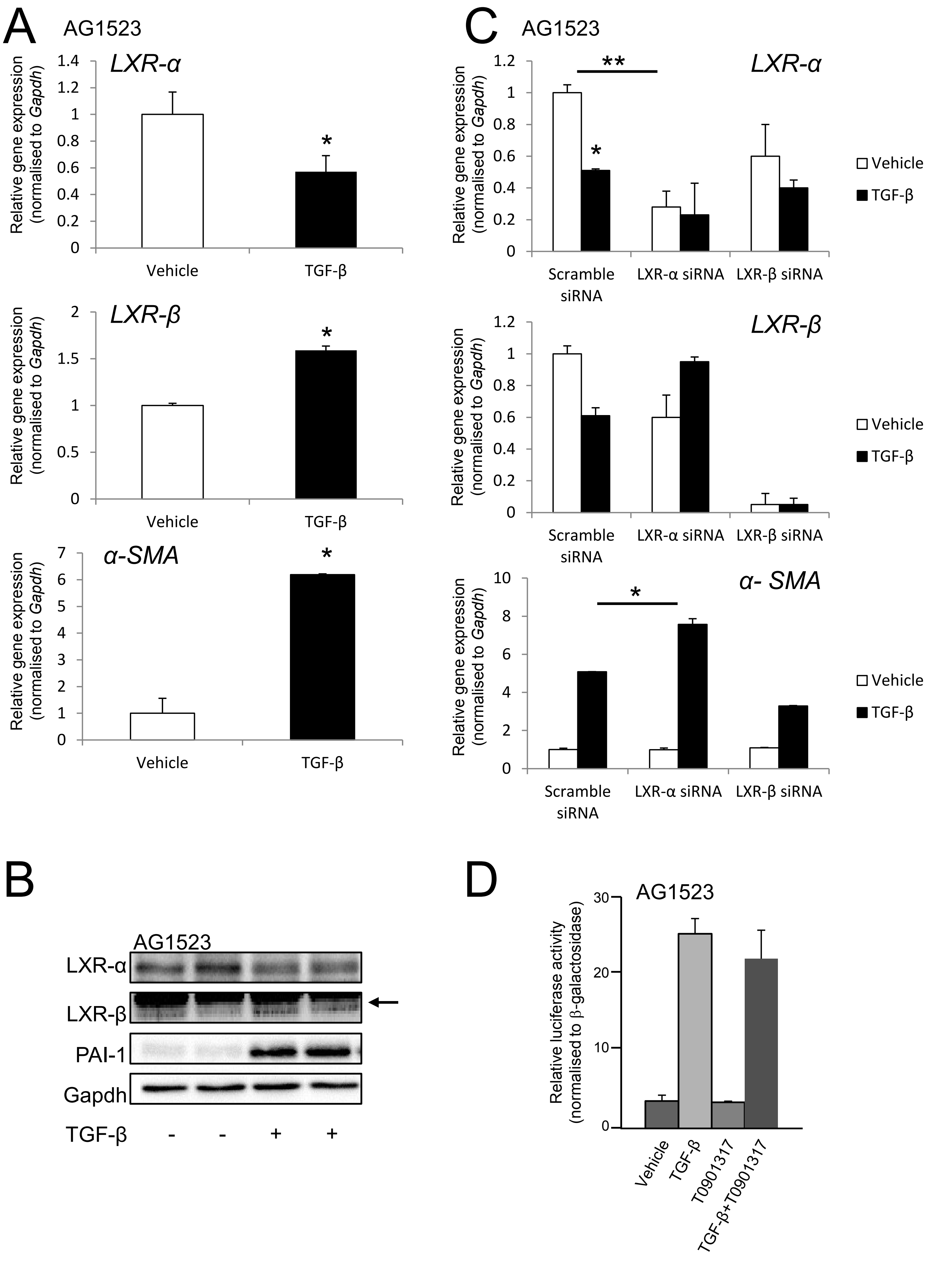

Figure S14. TGF-β regulates LXR expression and LXRα suppresses TGF-β-induced αSMA expression. (**A**) mRNA expression analysis of the three indicated genes in AG1523 cells in the absence or presence of 5 ng/ml TGF-β for 72 h, analyzed by real-time RT-PCR for each specific mRNA and normalised against the housekeeping *Gapdh* mRNA. The data are expressed as bar graphs of average determinations with corresponding standard errors from triplicate determinations. Stars indicate significant difference at $p<0.05$. (**B**) Protein expression analysis in AG1523 total cell lysates stimulated (+) or not (-) with 5 ng/ml TGF-β for 24 h. Immunoblots for the indicated proteins and for Gapdh, the protein loading control, are shown. An arrow marks the specific protein band. (**C**) mRNA expression analysis of the three indicated genes in AG1523 cells transfected with the indicated siRNAs and stimulated (black bars) or not (white bars) with 5 ng/ml TGF-β for 72 h, analysed by real-time RT-PCR as in panel A. (**D**) Luciferase activity assay in AG1523 cells transiently transfected with the $CAGA_{12}$-luc and CMV-β-gal reporters prior to stimulation with 5 ng/ml TGF-β or 10 μM T0901317 for 18 h. The luciferase activity was normalised to the corresponding β-galactosidase activity. The bar graph shows average values derived from triplicate determinations and their corresponding standard errors.
